# *Mycobacterium tuberculosis* resisters despite HIV exhibit activated T cells and macrophages in their pulmonary alveoli

**DOI:** 10.1172/JCI188016

**Published:** 2025-01-21

**Authors:** Monica Dallmann-Sauer, Vinicius M. Fava, Stephanus T. Malherbe, Candice E. MacDonald, Marianna Orlova, Elouise E. Kroon, Aurélie Cobat, Stéphanie Boisson-Dupuis, Eileen G. Hoal, Laurent Abel, Marlo Möller, Jean-Laurent Casanova, Gerhard Walzl, Nelita Du Plessis, Erwin Schurr

**Affiliations:** 1 Program in Infectious Diseases and Immunity in Global Health, The Research Institute of the McGill University Health Centre, Montreal, Canada.; 2 McGill International TB Centre, and; 3 Departments of Human Genetics and Medicine, Faculty of Medicine and Health Science, McGill University, Montreal, Canada.; 4 South African Medical Research Council Centre for Tuberculosis Research; Biomedical Research Institute, Division of Immunology, Department of Biomedical Sciences and; 5 South African Medical Research Council Centre for Tuberculosis Research; Division of Molecular Biology and Human Genetics, Faculty of Medicine and Health Sciences, Stellenbosch University, Cape Town, South Africa.; 6 St. Giles Laboratory of Human Genetics of Infectious Diseases, Rockefeller Branch, The Rockefeller University, New York, USA.; 7 Laboratory of Human Genetics of Infectious Diseases, Necker Branch, INSERM U1163, Necker Hospital for Sick Children, Paris, France.; 8 Université Paris Cité, Imagine Institute, Paris, France.; 9 Howard Hughes Medical Institute, New York, New York, USA.; 10 Pediatric Hematology and Immunology Unit, Necker Hospital for Sick Children, AP-HP, Paris, France.

**Keywords:** Infectious disease, Macrophages, T cells, Tuberculosis

## Abstract

**BACKGROUND:**

Natural resistance to Mycobacterium tuberculosis (*Mtb*) infection in some people with HIV (PWH) is unexplained.

**METHODS:**

We performed single cell RNA-sequencing of bronchoalveolar lavage cells, unstimulated or ex vivo stimulated with Mtb, for 7 PWH who were tuberculin skin test (TST) and IFN-γ release assay (IGRA) positive (called LTBI) and 6 who were persistently TST and IGRA negative (called resisters).

**RESULTS:**

Alveolar macrophages (AM) from resisters displayed a baseline M1 macrophage phenotype while AM from LTBI did not. Resisters displayed alveolar lymphocytosis, with enrichment of all T cell subpopulations including IFNG-expressing cells. In both groups, mycobactericidal granulysin was expressed almost exclusively by a T cell subtype that coexpressed granzyme B, perforin and NK cell receptors. These poly-cytotoxic T lymphocytes (poly-CTL) overexpressed activating NK cell receptors and were increased in resister BAL. Following challenge with *Mtb*, only intraepithelial lymphocyte-like cells from LTBI participants responded with increased transcription of IFNG. AM from resisters responded with a stronger TNF signature at 6 hours after infection while at 24 hours after infection, AM from LTBI displayed a stronger IFN-γ signature. Conversely, at 24 hours after infection, only AM from resisters displayed an upregulation of MHC class I polypeptide–related sequence A (MICA) transcripts, which encode an activating ligand for poly-CTL.

**CONCLUSION:**

These results suggest that poly-CTL and M1-like pre-activated AM mediate the resister phenotype in PWH.

**FUNDING:**

National Institutes of Health. Canadian Institutes of Health Research. Digital Research Alliance of Canada. French National Research Agency. French National Agency for Research on AIDS and Viral Hepatitis. St. Giles Foundation. General Atlantic Foundation. South African Medical Research Council Centre for Tuberculosis Research.

## Abstract 2

People with HIV who are resistant to infection with * Mycobacterium tuberculosis* display activated alveolar macrophages and increased numbers of alveolar mycobactericidal poly-cytotoxic T cells.

## Introduction

An estimated 7.5 million incident cases of tuberculosis (TB) were reported in 2022, making it the highest number of newly diagnosed cases since 1995 (1). Globally, 6.3% of incident cases were in people with HIV (PWH) (1). Relative to HIV-negative persons, PWH have a higher risk of developing clinical TB, making TB in PWH a major public health challenge in areas of high HIV prevalence (1–3). In Southern Africa, more than 50% of people who fell ill with TB in 2022 were PWH (1).

Exposure to *Mycobacterium tuberculosis* (*Mtb*), the cause of TB, leads to a spectrum of clinical manifestations ranging from absence of immunological or clinical features to life-threatening TB (4–6). Among exposed persons with no clinical symptoms, differences in innate immune response (7–9), *Mtb*-specific antibody production (10–14), IFN-γ–independent T cell responses (13–15), and *Mtb*-specific CD4^+^ T cell immunity (16, 17) indicate the complexity of *Mtb* infection control. The clinical and public health standards of established *Mtb* infection are provided by the tuberculin skin test (TST) and IFN-γ release assays (IGRA) (18). The 2 tests measure different aspects of CD4^+^ and CD8^+^ T cell–dependent immunity in the periphery (18, 19). People who despite documented exposure remain persistently negative in both assays are “resisters” to IFN-γ conversion (20–23), while those with positive tests and no clinical evidence of TB are diagnosed with “latent TB infection” (LTBI) (24).

The identification of persons who are resistant to establishment of *Mtb* infection is complicated by the need to quantitate exposure and the lack of tools for direct, early-stage detection of *Mtb* in the lung. Moreover, the assays used to infer infection are conducted in peripheral blood and provide readouts of unknown relevance for protective immune responses in the lung. However, the use of both tests does provide a high negative predictive value for risk of TB, an important aspect when studying infection resistance (25, 26). In epidemiological studies, test negativity indicates absence of *Mtb* infection; however, this does not imply absence of *Mtb* in alveoli of exposed persons. Test negativity in persons with high persistent exposure may reflect true *Mtb* infection resistance or in the context of shorter follow-up indicate early clearance,which is a relative measure of resistance and associated with innate immune correlates (27–30). Understanding host mechanisms that rule out early engagement of *Mtb* in the pulmonary alveoli and persistently negate establishment of a pulmonary *Mtb* infection is of critical importance to deriving interventions that prevent TB and transmission of *Mtb*.

To address this need in the context of HIV-TB, we enrolled PWH who during the pre–anti-retroviral therapy (pre-ART) era had documented low CD4^+^ cell counts, putting them at increased risk of TB. Following immune reconstitution by long-term ART, all study participants remained free of TB with a subset being IGRA/TST-negative despite being PWH and living in a region of high TB exposure (12). All participants underwent bronchoalveolar lavage (BAL), and we performed single-cell RNA sequencing (scRNA-Seq) with the resulting alveolar immune cells. We found striking differences in BAL fluid cell proportions and transcriptomic state at baseline and in response to *Mtb* between resisters and LTBI. BAL samples from resisters were highly enriched for lymphocytes including subpopulations of CD4^+^ and CD8^+^ tissue resident memory (TRM) T cells, and a subpopulation of mycobactericidal poly-cytotoxic T lymphocytes (poly-CTLs) (*GNLY/GZMB/PRF1*^hi^) expressing a suite of NK cell receptors. Resister alveolar lymphocytes presented higher counts of *IFNG* transcripts over cells from LTBI samples constitutively and after ex vivo *Mtb* challenge. Resister alveolar macrophage (AMs) showed a pronounced shift toward a classically activated M1 phenotype at baseline. At 24 hours after *Mtb* challenge, transcripts for MHC class I polypeptide–related sequence A (*MICA*) and its activating NK receptor natural killer group 2D receptor (NKG2D) (*KLRK1*) were strongly overrepresented in AM and in poly-CTLs of resisters, respectively. Combined, our data strongly support a key role of mycobactericidal poly-CTLs and activated AM cells in resistance to *Mtb* infection as determined by IGRA and TST in PWH.

## Results

### Cell-type distribution of BAL cells.

**Figure 1:**
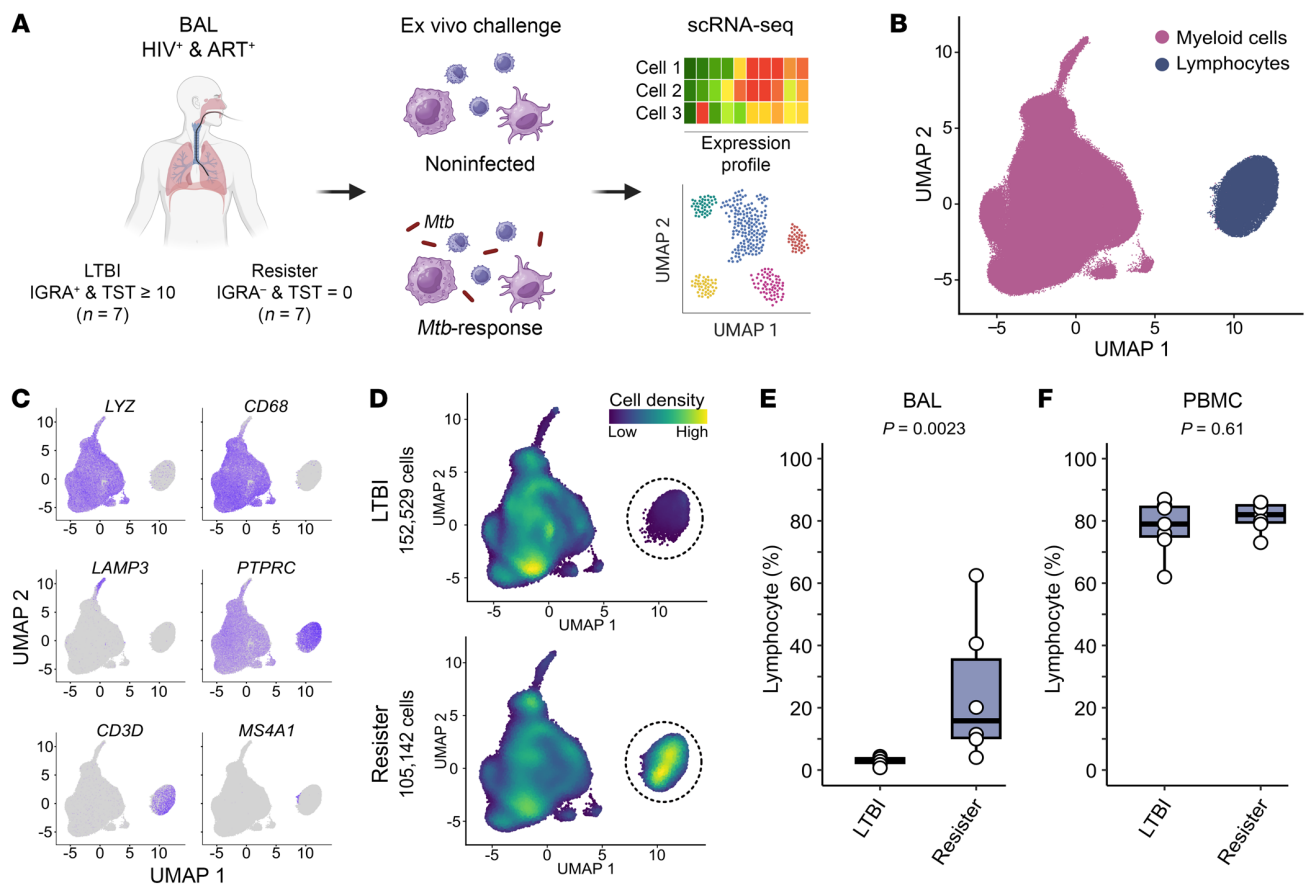
Resisters have higher lymphocyte proportions in cells obtained by BAL compared with LTBI. (**A**) Schematic representation of the study design. BAL cells were obtained from all study participants and scRNA-Seq was conducted at 6 hours and 24 hours in the presence and absence of *Mtb* infection. Gene-expression data were derived both for uninfected (defined as baseline) and infected BAL cells. Analysis of scRNA-Seq data was used to estimate BAL cell identities and proportions and to perform DE analysis. Created with BioRender.com. (**B**) UMAP of the scRNA-Seq data from the BAL cells of all subjects identified myeloid cells and lymphocytes as 2 main populations. (**C**) Gene expression of canonical markers for macrophages (*LYZ* and *CD68*), DCs (*LAMP3*), leukocytes (*PTPRC* [CD45]), T cells (*CD3D*), and B cells (*MS4A1*). Higher expressions are shown by darker colors in the UMAP. (**D**) Density of cells obtained from LTBI and resister participants. Dashed-line circles indicate the BAL lymphocytes in the 2 groups. Yellow and dark blue colors indicate the highest and lowest density of cells in the UMAP, respectively. UMAPs included samples irrespective of infection status and incubation time point. (**E**) Box plot of lymphocyte proportions (%) in BAL cells obtained from resister and LTBI participants. Each dot represents the average lymphocyte percentage obtained from the scRNA-Seq libraries per subject. (**F**) Lymphocyte proportion (%) in PBMCs for the same resister and LTBI participants.

Our study was restricted to PWH on long-term ART with controlled viral loads and no history of TB despite long-term exposure to *Mtb* in a high-transmission setting (Figure 1A). The 14 participants belonged to 2 well-defined phenotypic groups of equal size: participants classified as LTBI who tested IGRA positive and displayed a TST of 10 mm or more and participants coined resisters who persistently tested IGRA negative with a TST equal to 0 mm (Figure 1A and Table 1) (12). Active TB or other lung infections were excluded by chest X-ray and TB sputum testing. All participants agreed to undergo a BAL. On average we obtained 1.36 × 10^7^ (±1.85 × 10^6^) BAL cells in the resister group and 2.59 × 10^7^ (±1.32 × 10^7^) from the LTBI samples (*P* = 0.1649). The recovered cells were kept unstimulated or challenged with *Mtb* for 6 hours and 24 hours. We performed scRNA-Seq to investigate the BAL fluid cellular composition, gene expression levels in the absence of *Mtb*, and the transcriptomic responses to *Mtb* challenge (Figure 1A). After quality control resulting in exclusion of 1 resister, we obtained single-cell transcriptome results for 257,671 BAL cells from 6 resisters and 7 LTBI participants (Supplemental Table 1; supplemental material available online with this article; https://doi.org/10.1172/JCI188016DS1). Based on gene-expression profiles we found 2 main subsets of cells (Figure 1B). Innate immune cells including AMs and DCs constituted the largest subset, corresponding to 89% of the BAL cells, while the remaining 11% consisted of lymphocytes (T, B, and NK cells) (Figure 1, B and C). However, BAL cells comprised strikingly different proportions of myeloid and lymphoid cells between the 2 groups, where resisters presented a significantly higher proportion of lymphocytes (*P* = 0.002, Figure 1, D and E). While all LTBI subjects had less than 5% of lymphocytes (mean 2.93%) and more than 95% of myeloid cells (mean 97.07%) in their BAL samples, BAL samples from resisters presented a large spread of lymphocyte proportions ranging from 4% to 62.5% (mean 24.78%, Figure 1E and Supplemental Figure 1) with relatively lower proportions of myeloid cells (from 37.5% to 96%, mean 75.22%). We considered participants with BAL lymphocyte percentage of 10% or more to display alveolar lymphocytosis. None of the clinical or demographic variables collected correlated with the degree of lymphocytosis. We noted minor peripheral blood contamination in the BAL fluid of 3 samples from both groups, which had no correlation with lymphocytosis (Supplemental Table 1). We also obtained PBMCs from the same participants and found no significant differences in lymphocyte proportions (*P* = 0.61, Figure 1F) or cell subpopulations in PBMCs between the 2 groups (Supplemental Table 2). These results showed that alveolar lymphocytosis observed in resisters was not connected with lymphocyte counts in peripheral blood.

### Characteristics of alveolar myeloid cells in the absence of ex vivo Mtb challenge.

**Figure 2:**
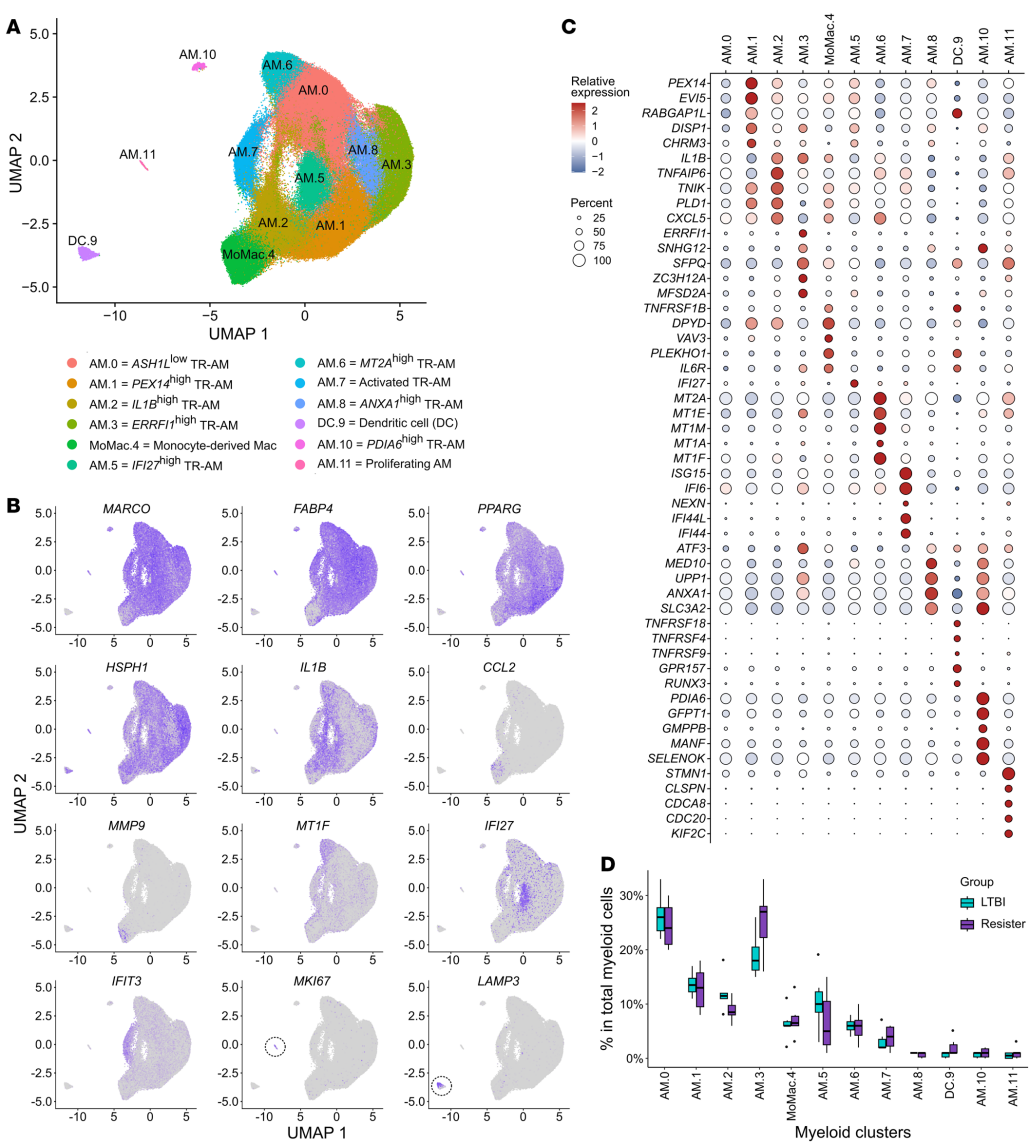
Annotation of myeloid cell subpopulations in BAL. (**A**) UMAP of the myeloid subset with 12 clusters and their annotations. TR, tissue resident. (**B**) UMAP showing the gene expression of selected canonical markers used for the annotation of the myeloid cell subpopulations. Higher expressions are denoted by darker colors. UMAPs included data from all samples and all conditions. (**C**) Top 5 genes with highest DE compared with the remaining myeloid cells for each cluster. Color and size correspond to the scaled expression and the percentage of cells expressing the gene by cluster, respectively. Data from noninfected samples. (**D**) Cluster proportions relative to the total myeloid population from resister and LTBI BAL samples. Black dots represent the outliers from the ±1.5× interquartile range. Data from 6-hour noninfected samples.

To better define the differences in BAL cell subpopulations between resister and LTBI samples, we clustered the myeloid and lymphoid cells separately. Clustering was done with all the infected and noninfected samples and the 2 time points. In the myeloid subset, we identified 12 clusters (Figure 2A and Supplemental Table 3). Of these, 1 small cluster (DC.9) consisted of DCs, while all remaining clusters were subpopulations of macrophages (Figure 2, A–C). All macrophages expressed markers that were consistent with tissue-resident AM (*MARCO*, *PPARG*, *FABP4*) except for cluster MoMac.4, which we annotated as infiltrating monocyte-derived macrophages (*CCL2*, *CSFR1*, *MMP9*, and CD14) (Figure 2B and Supplemental Figure 2). Next, we proceeded to investigate the differential profile of the myeloid cells between resister and LTBI cells in the absence of the *Mtb* ex vivo challenge. To compare the baseline difference in subpopulation proportions between the groups, we determined, for each participant, the percentage of each cluster relative to their total myeloid cell count from the 6-hour noninfected samples (Figure 2D). Two clusters presented higher proportions in resister samples with nominal significance using a 2-sided Wilcoxon’s test (AM.3 *P* = 0.041 and DC.9 *P* = 0.026), but failed to pass multiple test correction (Bonferroni’s threshold: *P* < 0.0042).

**Figure 3:**
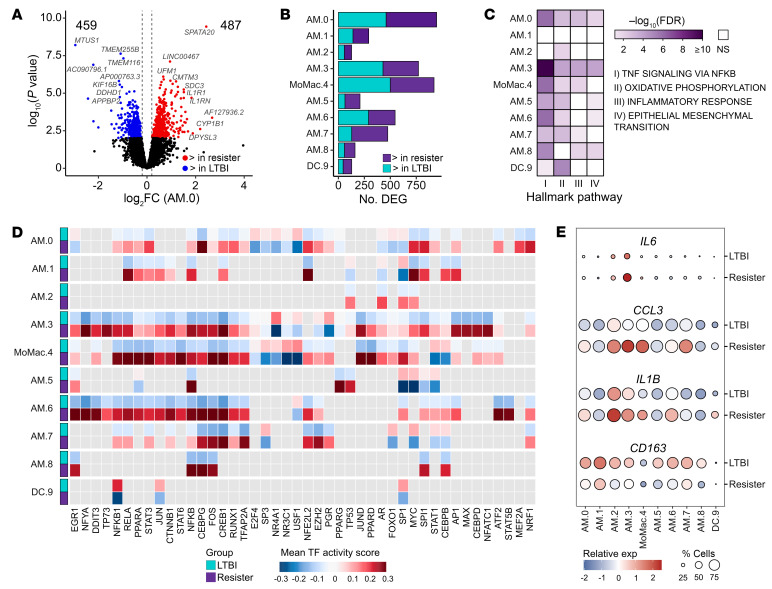
Gene-expression differences in the absence of *Mtb* between resister and LTBI myeloid cell subpopulations in the absence of *Mtb*. (**A**) Volcano plot for differences in gene expression between resister and LTBI samples for subpopulation AM.0. Volcano plots of remaining clusters are shown in Supplemental Figure 3. Dashed lines correspond to the log_2_FC thresholds of –0.2 and 0.2. Total numbers of DEG higher (red) or lower (blue) expressed in resister samples are indicated in the top corners. (**B**) Numbers of DEG across all myeloid cell subpopulations. Purple and light blue indicate DEG with higher or lower expression in cells from resisters. (**C**) Selected Hallmark pathways enriched for genes with higher expression in resister compared with LTBI cells. (**D**) Differential TF activity in resister and LTBI BAL samples for 6-hour noninfected cells. The heatmap shows the top 10 TF displaying the largest mean differential activity per myeloid cell subpopulation, except for clusters with less than 10 significant TF. The mean TF activity scores for cells in each cluster are shown for the LTBI and resister cells. Positive and negative scores indicate stronger or weaker/inactive TF activity, respectively. Nonsignificant (FDR > 0.01) or not tested TFs are shown in gray. (**E**) Gene expression of selected M1 genes *IL6*, *CCL3*, and *IL1B*, and the M2 gene *CD163* in 6-hour noninfected cells. Color and size correspond to the scaled expression and the percentage of cells expressing the gene by cluster, respectively.

To better understand the transcriptomic profiles of these myeloid BAL cell populations, we performed pseudobulk differential expression (DE) analysis between cells from resister and LTBI participants. The DE analyses were done for each cluster independently, excluding AM.10 and AM.11 due to their low number of cells per library. For the 9 AM clusters and the DC cluster, we detected a total of 4,275 genes (comprising 2,167 distinct genes) that were differentially expressed between resister and LTBI cells (Figure 3, A and B, Supplemental Figure 3, and Supplemental Table 4). Next, we performed a gene-set enrichment analysis (GSEA) to determine which Hallmark pathways were enriched in genes differently expressed at baseline between the resister and LTBI cells. Strikingly, pathway gene sets were enriched mostly among genes with higher expression in resister compared with LTBI cells (Supplemental Table 5). For example, genes from TNF signaling via NF-κB, oxidative phosphorylation, and inflammatory response pathways were enriched among genes more expressed in resister AM compared with LTBI, with the most pronounced enrichment in AM.3 (*ERRFI1*^hi^ TR-AM) cells (Figure 3C). The differentially expressed pathways at baseline are consistent with an important role of metabolic state and TNF signaling in the resister phenotype and TB susceptibility.

Next, we investigated the extent to which differential baseline gene expression reflected changes in transcription factor (TF) activities. TF activity was inferred from the gene expression of target genes induced or repressed by the TFs. For the TF regulatory network analysis, we calculated TF activity scores using the genes differently expressed between resisters and LTBI samples in the absence of *Mtb* (Figure 3D and Supplemental Table 6). In AM, we found significant differences in TF activities between the groups for TFs involved in M1 macrophage polarization (FDR < 0.01), for example, TFs AP1, NF-κB, CEBPG, and IRF1 that are linked to an M1 state showed stronger activity in AM from resisters (Figure 3D and Supplemental Table 6). Similarly, we found higher expression of M1 genes such as *IL6*, *CCL3*, and *IL1B* as well as lower expression of the canonical M2 marker *CD163* in AMs from resister compared with LTBI samples (Figure 3E). This showed that AMs from resisters were shifted toward an M1 transcriptomic profile in the absence of *Mtb*.

To investigate whether the differences in the transcriptomic profile of resister and LTBI alveolar myeloid cells at baseline were linked with the difference in alveolar lymphocyte proportions between the groups, we repeated the baseline comparison of resister versus LTBI in myeloid cells adding BAL lymphocyte proportion as a covariate in the model (Supplemental Figure 4). Differentially expressed genes (DEG) that remained significant in this analysis were independent from alveolar lymphocytosis in resisters. The adjustment on lymphocyte proportion differentially affected the myeloid cell subpopulations (Supplemental Figure 4). More strikingly, while we still observed DEG between resister and LTBI cells, the number of DEG was smaller, resulting in a substantially smaller number of pathways enriched among the DEG (Supplemental Figure 4). This suggested that most of the myeloid cell baseline functional transcriptomic differences observed between resisters and LTBI were associated with the alveolar lymphocytosis.

### Characteristics of alveolar lymphoid cells in the absence of Mtb.

**Figure 4:**
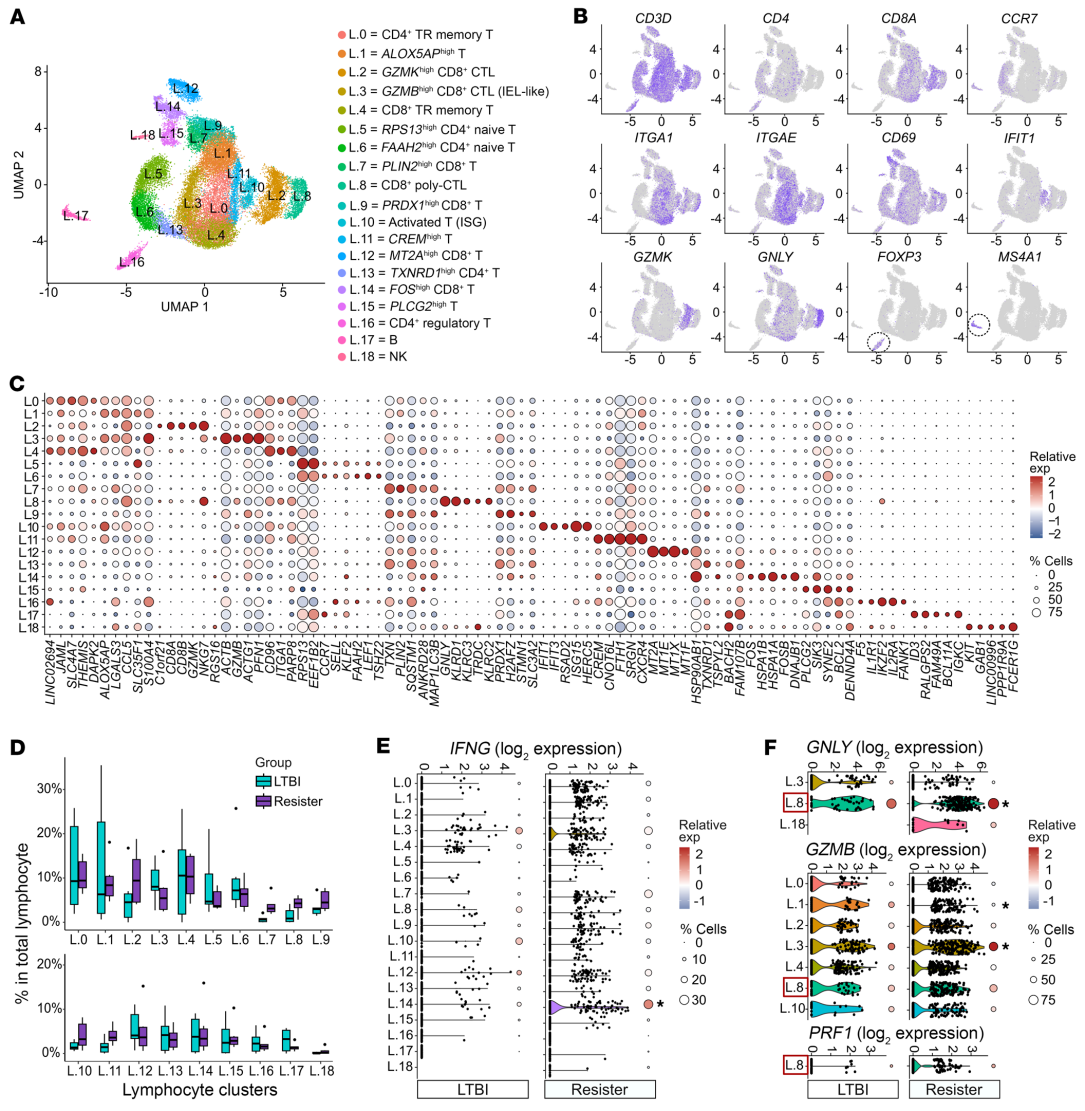
Annotation of lymphocyte subpopulations in BAL. (**A**) UMAP of the lymphocyte subset showing 19 clusters and their annotations. Data from both groups with samples from all conditions. (**B**) Gene expression of selected canonical markers used for the annotation of lymphocyte clusters. Higher expression is reflected by darker colors. (**C**) Top 5 genes with higher expression for each cluster compared with the remaining lymphocytes. Color and size correspond to the scaled expression and the percentage of cells expressing the gene by cluster, respectively. Data from noninfected samples. (**D**) Lymphocyte cluster proportions relative to total alveolar lymphocytes from resister and LTBI samples. Black dots represent the outliers from the ±1.5× interquartile range. Data from 6-hour noninfected samples. Two clusters presented nominal *P* < 0.05 using a 2-sided Wilcoxon’s test (L.7 *P* = 0.008 and L.11 *P* = 0.045) but failed to pass multiple test correction (Bonferroni’s threshold: *P* < 0.0026). (**E** and **F**) Gene expression of antimycobacterial mediators (**E**) *IFNG*, (**F**) *GNLY*, *GZMB*, and *PRF1* in 6-hour noninfected lymphocytes from LTBI and resisters. Each dot in the violin plots represents a cell. The color and size legend of the circles on the right of each violin as detailed in panel **C**. The asterisks indicate a significant gene expression difference between resister and LTBI clusters (Wilcoxon’s *P* < 0.05). The L.18 cluster showing less than 10 cells in LTBI is not plotted. In **F**, only the clusters presenting 25% or more of positive cells in at least one of the groups are shown and the only cluster coexpressing the 3 genes is indicated with a box (L.8).

Next, we annotated the subpopulations in the lymphocyte subset, where we identified 19 clusters (Figure 4A, Supplemental Figure 2, and Supplemental Table 3). The majority of lymphocyte clusters comprised T cells (CD3^+^), including CD4^+^ naive T cells (*CCR7*, *SELL* [CD62L]), CD4^+^ regulatory T cells (*FOXP3*, *CTLA4*), CD8^+^ CTLs (*GZMs*), and CD4^+^ and CD8^+^ TRM cells expressing tissue-resident markers (Figure 4, A–C, Supplemental Figure 2, and Supplemental Table 3). We also detected one cluster of NK cells (*KLRC2*, *NCAM* [CD56]) and 1 B cell cluster (*MS4A1*, *CD79* and CD19) (Figure 4, A–C, and Supplemental Figure 2).

Given the significantly higher counts of lymphocytes in all clusters from resister BAL samples (Figure 1E), we then asked if the lymphocyte make-up differed between the 2 groups. Hence, we analyzed the difference in the proportions of the resister and LTBI lymphocytes in the absence of the *Mtb* ex vivo challenge. There were no significant differences among cluster proportions of total lymphocytes between the 2 groups (Figure 4D) and no group differences in the ratio of CD4^+^ to CD8^+^ T cells (Supplemental Table 2). While there was a large spread of lymphocyte cluster proportions in the LTBI group, this was a result of the small cell counts in those samples.

We then asked whether lymphocytosis resulted from the disproportionate contributions of specific lymphocyte clusters to overall BAL cell counts. We observed that while clusters were enriched in resister BAL fluid relative to LTBI at different rates, these differences did not explain overall lymphocytosis, as all clusters were enriched in resister BAL fluid (Supplemental Figure 5). Hence, all lymphocyte subpopulations were contributing to the alveolar lymphocytosis in the resister group.

We then compared the baseline transcriptomic profile of BAL lymphocytes from resister to LTBI BAL samples. The low T cell counts in LTBI BAL fluid precluded the use of a comprehensive pseudobulk DE analysis at the level of the 19 lymphocyte clusters. Hence, we compared the transcript expressions of resister and LTBI cells for 6-hour noninfected lymphocytes at the single-cell level. In this single-cell–driven analysis we focused on genes that are known to be linked to antimycobacterial host responses, T cell activation and cytotoxicity, and lymphocyte tissue retention (Figure 4, E and F, and Supplemental Figure 6). The key role of IFN-γ in the induction of M1 polarization of macrophages and the antimycobacterial immunity has been unambiguously established (31–33). In resisters, the clusters with the largest proportion of *IFNG*-positive cells were 3 subpopulations of CD8^+^ T cells: L.3 (*GZMB*^hi^CD8^+^ CTL), which presented a profile of intraepithelial lymphocytes (intraepithelial lymphocyte–like (IEL-like) cells), L.7 (*PLIN2*^hi^ CD8^+^ T), and L.14 (*FOS*^hi^ CD8^+^ T cell) (Figure 4E). Of these, the resister L.14 cluster displayed significantly higher levels of *IFNG* in a larger proportion of cells compared with the LTBI L.14 cluster (Figure 4E). In addition, despite the overall similar *IFNG* expression levels in the remaining clusters between resister and LTBI samples, the numbers of *IFNG*-positive cells per BAL sample were markedly higher in resisters as a direct result of lymphocytosis in the latter group (Figure 4E). These results suggested higher constitutive exposure of resister AM to secreted IFN-γ consistent with the M1-like polarization transcriptomic profile of the resister AM (Figure 3, C and D) and its association with the BAL lymphocyte proportion (Supplemental Figure 4).

We determined the transcript counts of 9 genes involved in CTL cytotoxicity, including the antimicrobial effector molecules granulysin (*GNLY*), granzyme B (*GZMB*), and perforin (*PRF1*) (Figure 4F and Supplemental Figure 6A). *GNLY*, *GZMB*, and *PRF1* are key effectors of T cell mycobactericidal immunity (34–37). We found a lower and higher *GZMB* expression in resister L.1 and L.3, respectively, compared with the same clusters in the LTBI samples (Figure 4F). Moreover, we found higher expression of *GZMA* and *GZMH* in resister cells compared with LTBI in 1 and 4 clusters, respectively (Supplemental Figure 6A). Coexpression of *GNLY*, *GZMB*, and *PRF1* genes has been shown to function synergistically to kill intracellular mycobacterial pathogens (36, 37), and we detected 1 cluster, L.8, coexpressing the 3 genes at baseline (Figure 4F). Since these cells were CD3 and CD8 positive, we annotated the L.8 cluster as CD8^+^ poly-CTLs (Figure 4B and Supplemental Figure 2). In L.8, *GZMB* and *PRF1* were expressed at approximately the same level in cells from the resister and LTBI participants, while *GNLY* was detected with higher expression in the resister cells (Figure 4F). Moreover, while 3′ scRNA-Seq may not be a sensitive approach to detect T cell receptor (TCR) type expression, we did notice that of all T cell subpopulations, L.8 cells expressed the highest transcript counts for TCR γ and δ constant regions while also expressing TCR α and β constant regions as well as NK receptors (Figure 4, B and C, and Supplemental Table 3). Finally, we investigated the expression of genes involved in lymphocyte tissue retention. The CD4^+^ and CD8^+^ TRM and the CD8^+^ IEL-like cells presented the highest expression levels of the 4 tissue resident markers (*ITGA1* [CD49a], *ITGAE* [CD103], *CXCR6*, and *CD69*) with no consistent difference between resister and LTBI cells (Supplemental Figure 6B). In the remaining clusters, we noted a trend of higher expression of tissue-resident markers in the resister T cell clusters with the highest proportion fold enrichment in resister BAL fluid compared with LTBI (Supplemental Figure 6B). For example, in L.7 and L.8, CD8^+^ poly-CTL tissue retention genes were detected only in the resister cells (Supplemental Figure 6B). These results suggested higher tissue retention of these cells in resister alveoli.

### Alveolar myeloid cell response to ex vivo Mtb challenge.

**Figure 5:**
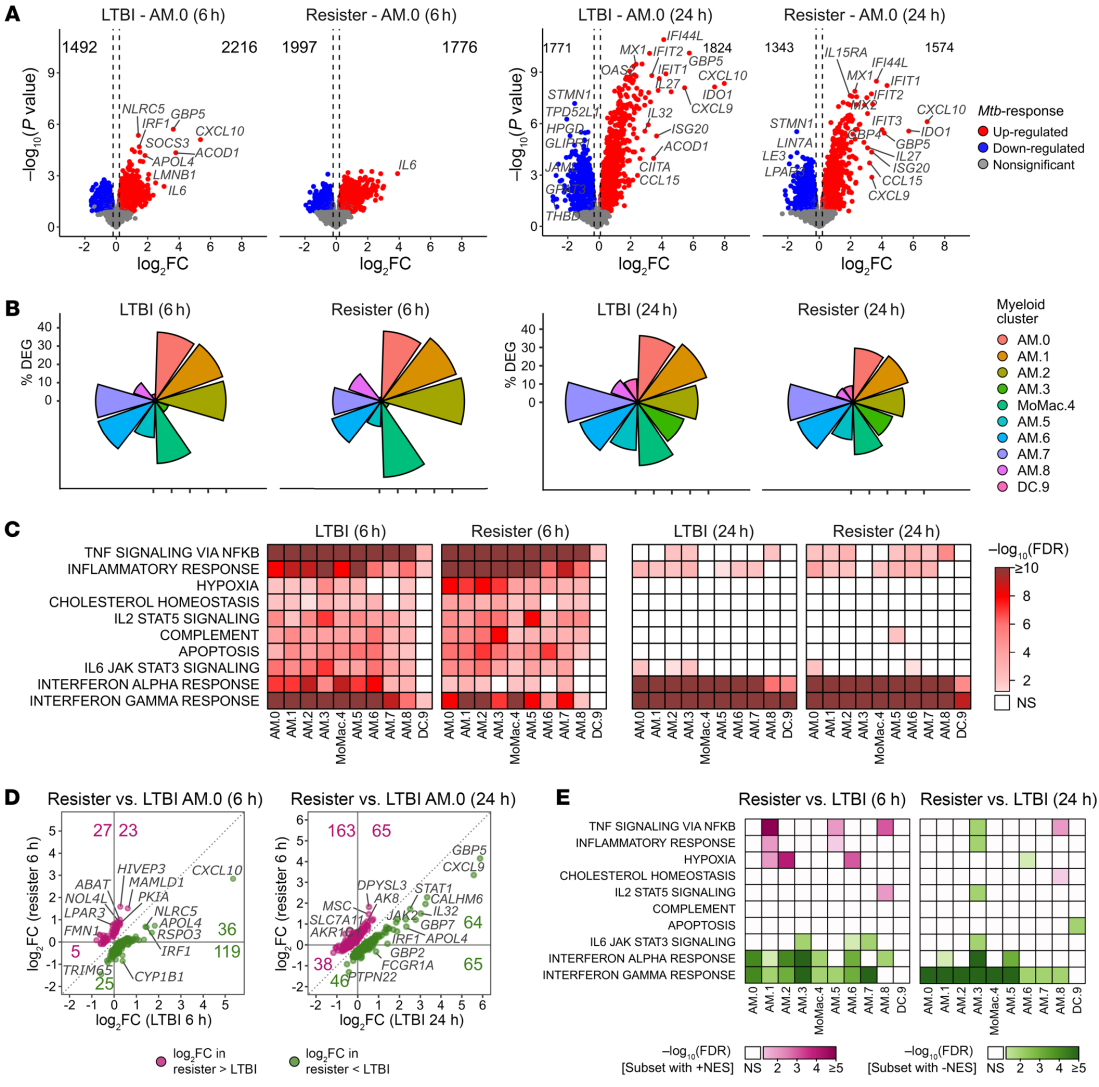
AM and DC responses to ex vivo infection with *Mtb* in resister and LTBI cells. (**A**) Volcano plots of differential gene expression in response to *Mtb* challenge by group and time after infection for subpopulation AM.0. Dashed lines correspond to the log_2_FC thresholds of –0.2 and 0.2. Total numbers of up- and downregulated DEG are indicated in the top corners (FDR < 0.2). (**B**) Proportions of DEG per cluster in response to *Mtb* challenge by group and time point. (**C**) GSEA results for selected Hallmark pathways that display enrichment for genes with changed expression in response to *Mtb* across clusters. All significant pathways presented in this figure were enriched for upregulated genes. Nonsignificant results (FDR > 0.05) are shown in white. (**D**) Log_2_FC of expression for cluster AM.0 genes that respond significantly differently to *Mtb* in resister (*x* axis) and LTBI (*y* axis) cells. The coordinate lines correspond to log_2_FC = 0. For each section of the plot, the total number of DEG is presented. (**E**) GSEA for Hallmark pathways based on the significantly differential *Mtb* response between resisters and LTBI at 6 hours and 24 hours. Genes were ranked according to overresponse in resister samples. Hence, positive and negative NES correspond to enrichment of genes with higher and lower log_2_FC in resister compared with LTBI cells, respectively. Hallmark pathways as in panel **C**.

Next, we compared the gene expression of *Mtb*-challenged samples from 6 hours and 24 hours after infection against the corresponding noninfected samples by group (Figure 5, A and B, and Supplemental Tables 7 and 8). In both groups, upregulated genes at 6 hours implicated a range of immune-mediated and inflammatory pathways with the strongest enrichment being the TNF signaling via NF-κB pathway, while at 24 hours, the transcriptomic changes were focused on interferon response pathways (Figure 5C and Supplemental Table 9). Then, we formally compared the transcriptomic *Mtb* responses between resister and LTBI cells by interaction analysis to identify genes with significantly greater magnitude of changes (from the noninfected to the *Mtb*-challenged cells) in the resister group compared with the changes in the LTBI group (Figure 5D and Supplemental Tables 7 and 8). At 6 hours after infection, the oxidative phosphorylation pathway was enriched among the genes more strongly downregulated in the resister macrophages compared with LTBI (Supplemental Tables 9 and 10). At the same time point, we found TNF signaling via NF-κB, inflammatory response, and hypoxia pathways enriched among genes with a stronger upregulation in resister compared with LTBI clusters (Figure 5E and Supplemental Table 10). Conversely, the interferon γ response and the interferon α response pathways were significantly enriched among genes more strongly upregulated in LTBI samples at both time points, albeit this was most pronounced for IFN-γ signalling at the 24-hour time point (Figure 5E and Supplemental Table 10). The stricter control of IFN-γ signalling in resister cells is likely a result of the homeostatic adaption of these cells to the significantly higher constitutive levels of IFN-γ (Figure 4E).

### Alveolar lymphoid cell response to ex vivo Mtb challenge.

**Figure 6:**
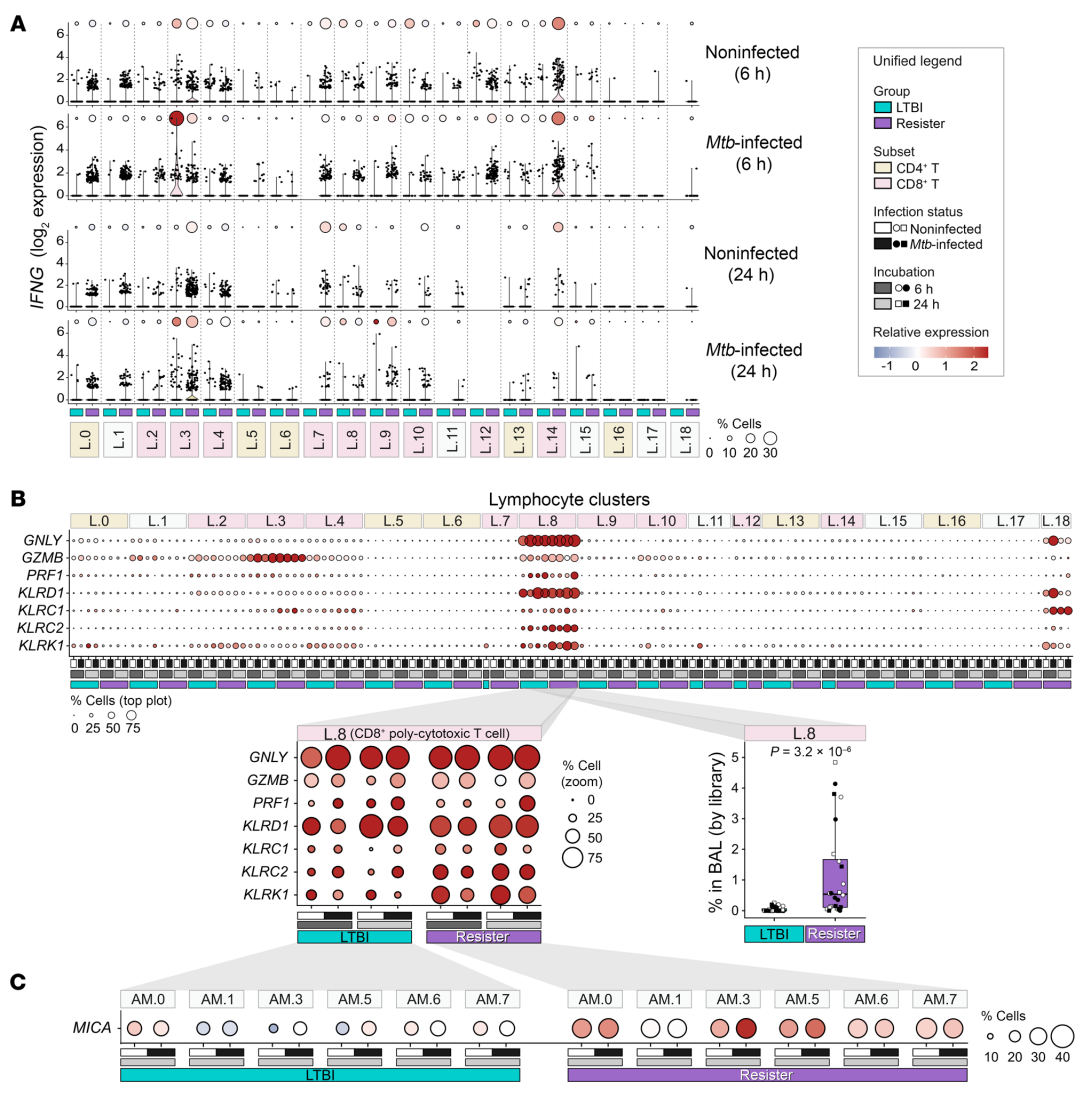
Alveolar lymphocyte responses to ex vivo infection with *Mtb*. (**A**) Kinetics of *IFNG* transcription at 6 hours and 24 hours in presence and absence of *Mtb* in LTBI and resister lymphocyte clusters. The violin plots present the density and distribution of the *IFNG* log_2_ expressions. The size of the circles in the dot plots indicates the percentage of cells expressing *IFNG*. Circle colors indicate *IFNG* scaled expression of the *IFNG*-positive cells. Each dot in the violin plots represents a cell. (**B**) Expression of antimicrobial effector molecules *GNLY*, *GZMB*, and *PRF1* and NK receptors *KLRD1*, *KLRC1*, *KLRC2*, and *KLRK1* at 6 hours and 24 hours of in vitro culture in presence and absence of *Mtb* in LTBI and resister lymphocyte clusters. The dot plot breakout insert focuses on 7 key cytotoxicity genes in cluster L.8. To the right of the breakout is a box plot of estimates of the L.8 cell frequencies in BAL samples including Wilcoxon’s *P* value for sample difference (Supplemental Figure 5). (**C**) *MICA* transcript expression at 24 hours of in vitro culture in presence and absence of *Mtb* in selected AM clusters from resister and LTBI participants.

Given the low cell counts in LTBI lymphocyte clusters, we used the same approach as for the baseline expression comparison of lymphocytes. We investigated the gene expression of key genes at the level of the 19 lymphocyte subpopulations (Figure 6, A and B, Supplemental Figure 7, and Supplemental Table 11). Only LTBI L.3 CD8^+^ IEL-like cells increased *IFNG* expression in terms of proportion of positive cells and expression levels in response to *Mtb* (Figure 6A). While not responding to *Mtb* challenge, at 6 hours, L.14 resister cells still presented the highest *IFNG* expression both in proportion of positive cells and expression levels of all clusters. Hence, as in the periphery, resister T cells did not mount an *IFNG* response to *Mtb*. However, the constitutively higher numbers of *IFNG*-expressing T cells in resister BALs, due to alveolar lymphocytosis, were maintained even after *Mtb* challenge (Figure 6B).

A main interest for our analyses were the *Mtb*-triggered expression changes of the antimicrobial effector molecules *GNLY*, *GZMB*, and *PRF1*. Irrespective of *Mtb* challenge, only the CD8^+^ poly-CTLs from cluster L.8 coexpressed all 3 genes at high expression levels (Figure 4F and Figure 6B). In L.8 cells from resister and LTBI samples, *GNLY* was induced to similar levels in both groups by *Mtb* infection (Figure 6B). Similarly, *GZMB* was expressed at approximately the same level at 6 hours and 24 hours after infection in resister and LTBI L.8 cells (Figure 6B). Perforin showed a trend for higher expression in LTBI samples at 6 hours after *Mtb* challenge. However, at 24 hours, *PRF1* was expressed at the same level in a larger proportion of resister cells (Figure 6B). CD8^+^ poly-CTLs from L.8 also expressed the genes for the NK activating receptors NKG2D (*KLRK1*) and NKG2C (*KLRC2*) and for the inhibitory receptor NKG2A (*KLRC1*) as well as for CD94 (*KLRD1*), required for the CD94/NKG2C and CD94/NKG2A complexes (Figure 4C and Supplemental Table 3). The expression of these NK receptors can confer an innate-like cytotoxicity to CD8^+^ T cells (38, 39).

We explored the NK receptor transcription levels in L.8 following *Mtb* challenge (Figure 6B). *KLRD1* was expressed at approximately the same level at 6 hours and 24 hours after infection in both groups. Similarly, the *KLRC1* gene encoding the inhibitory NKG2A receptor was expressed at approximately the same low level at 6 hours and 24 hours after *Mtb* infection in both groups (Figure 6B). Conversely, the genes encoding the activating receptors *KLRC2* and *KLRK1* were expressed at higher levels in a larger proportion of L.8 cells by resisters. This was most pronounced for *KLRK1*, where at 24 hours after infection, more than 60% of L.8 cells in resisters expressed the gene versus only 20% in LTBI cells (a 3-fold difference, Figure 6B). Overall, the ratios of activating and inhibitory receptors demonstrated a strong switch in favor of activation of the CD8^+^ poly-CTLs in resisters. Even more striking, the L.8 cells were among the T cell clusters with the highest fold proportional enrichment in BAL from resister compared with LTBI samples (Supplemental Figure 5). The mean ratio of the CD8^+^ poly-CTLs was 0.046% of all BAL cells for the LTBI group and 1.2% for the resister group, presenting an over 26-fold increase in this group over LTBI (*P* = 3.2 × 10^-6^, Figure 6B and Supplemental Figure 5). The finding that 3 times as many cells express the *KLRK1* gene in resisters pointed to an on average approximately 50-fold higher number of *KLRK1*-positive L.8 cells in the resister BAL fluid (*P* = 1.5 × 10^-6^).

The heterodimers NKG2A-CD94 (*KLRC1* + *KLRD1*) and NKG2C-CD94 (*KLRC2* + *KLRD1*) interact with major histocompatibility complex, class I, E (HLA-E), while NKG2D (*KLRK1*) interacts with the nonclassical MHC class I ligands MICA and MICB (40, 41). In our data, *HLA-E* was highly expressed in all myeloid cell subpopulations and *HLA-E* expression was significantly induced by 24 hours of *Mtb* challenge to a similar extent in both groups *(*Supplemental Figure 8A). *MICA* and *MICB* genes were transcribed by macrophages with higher expression at the 24 hours after infection time point (Supplemental Figure 8B). *MICB* presented lower expression than *MICA* with similar levels by both groups. Conversely, at 24 hours. *MICA* expression was significantly upregulated in response to *Mtb* in 6 AM clusters from resisters while corresponding AM from the LTBI group showed substantially weaker upregulation of this genes (Figure 6C). For example, MICA upregulation in LTBI AM.3 (log_2_ fold change [log_2_FC] = 0.57; FDR = 0.03) was significantly weaker (interaction FDR = 0.19) than upregulation in resister AM.3 (log_2_FC = 1.15; FDR = 3.7 × 10^-5^) (Supplemental Table 8). Combined, these results supported a strong NKG2D (*KLRK1*)–MICA receptor ligand interaction in resister alveoli as a critical feature for recognition of *Mtb-*infected AM by CD8^+^ poly-CTLs.

## Discussion

The present single-cell transcriptomic study was carried out with BAL cells obtained from *Mtb* resisters among PWH (12). We uncovered that the resisters enrolled in our study displayed variable degrees of airway lymphocytosis. While the alveolar lymphocyte proportions in the resister samples were high, they fit within the upper distribution observed in BAL fluid for the general population in Cape Town, both in PWH (15.4 ±15.6% SD) and HIV-negative subjects (13.5 ±20.6% SD) (42). An extensive spread of alveolar T cell proportions has also been described in other surveys (43). Hence, our results support a key role of alveolar lymphocytes for TB pathogenesis that has so far received scant attention.

Most alveolar lymphocytes in our data were T cells that were grouped into 17 distinct clusters based on their transcriptomic make-up. All T cell clusters were enriched in resister compared with LTBI alveoli. Alveolar T cells included a poly-cytotoxic T cell cluster, annotated as L.8, that coexpressed 3 key molecules crucial for the immune response against *Mtb*: perforin (*PRF1*), granzyme B (*GZMB*), and granulysin (*GNLY*). These poly-CTLs kill a range of intracellular parasites (44) and effectively restrict *Mtb* growth (45). Recognition of *Mtb*-infected cells by poly-CTLs triggers degranulation and release of cytotoxic molecules in immune synapses. Perforin creates holes in the plasma membrane, facilitating the entry of granzyme B and granulysin into the host cells (46). Granulysin kills *Mtb* by altering the membrane permeability of the bacillus (35, 37, 47). Granulysin also delivers the protease granzyme B to intracellular bacteria, and their collaboration results in rapid bacterial death across multiple species (34, 48). In the macaque model of TB, CD8^+^ T cells expressing *PRF1*, *GZMB*, and *GNLY* were associated with protective granuloma (49) and linked with protection from *Mtb* in the early stage of infection (50). In humans, CD8^+^ poly-CTLs were linked to control of bacterial dissemination in leprosy (36, 48) and treatment success in TB (51, 52). Moreover, anti-TNF therapy was associated with the selective depletion of CD8^+^ poly-CTL and decreased antimicrobial capacity of PBMCs against Mtb (53).

Poly-CTLs of the L.8 cluster comprised a pool of CD8^+^αβTCR^+^ and a smaller proportion of double-negative γδTCR^+^ cells, where both subsets expressed the 3 cytotoxic molecules (*PRF1*, *GZMB*, and *GNLY*) as well as NK receptors. The latter included the activating *KLRC2* (NKG2C) and *KLRK1* (NKG2D) receptors that recognize HLA-E and MICA/B, respectively, as ligands on target cells. Interestingly, while NKG2C and NKG2D can work as costimulatory signals for TCR activation in T cells (54, 55), they can also induce the T cell effector function in a TCR-independent manner (36, 39, 56, 57). Hence, NKG2C^+^ and NKG2D^+^ T cells possess an innate-like function in early local protection against infection in the absence of antigen-specific responses. Of the 2 receptor genes, *KLRK1* was expressed by an approximately 3-fold higher proportion of L.8 resister cells compared with the same cluster in LTBI, highlighting the role of an NKG2D-dependent activation of alveolar mycobactericidal poly-CTLs in resisters.

Among the alveolar T cells, we also identified T cells resembling CD4^+^ TRM (L.0) and CD8^+^ (L.4) TRM as well as IEL-like cells (L.3). TRM and IEL cells are located at pathogen entry portals and persist locally at mucosal and epithelial tissue sites where they provide defense against pathogens such as *Mtb* (58–60). On site, TRM cells are poised to deliver a fast and robust response upon exposure to a pathogen and promote the generation of antibodies (39, 61). In fact, a subpopulation of CD4^+^ TRM cells that are colocalized with B cells in inducible bronchus-associated lymphoid tissue (iBALT) promote local antibody production and enhance CD8^+^ TRM cells via IL-21 signaling (62, 63).

The resister and LTBI phenotype are defined by the absence or presence of IFN-γ adaptive T cell immunity against mycobacterial antigens in the periphery. In alveolar cells, only IEL-like L.3 cells from LTBI responded with the expression of *IFNG* in response to ex vivo challenge with *Mtb*. However, while *IFNG* transcript counts in resister T cell clusters remained constant after *Mtb* challenge, they remained many times higher than in LTBI T cell clusters. This suggested that constitutively high IFN-γ levels in alveoli are a key aspect of the resister phenotype. IFN-γ production in alveoli can activate AM and generate innate memory AM with increased microbicidal activity (64–66). A similar effect in resister alveoli is supported by the finding that resister AM presented an M1-activation phenotype while LTBI AM did not.

The comprehensive analysis of the AM transcriptomic response to ex vivo *Mtb* challenge revealed a temporal biphasic response in both phenotypic groups. At 6 hours after infection, the changes were dominated by TNF signaling and response to hypoxia. This TNF-dominated response was significantly stronger in resister AM (Figure 5E), consistent with the stronger TNF signaling observed in peripheral monocytes from HIV-negative resisters (8). TNF has been long associated with increased microbicidal activity, including the production of ROS via remodeling of the NADPH oxidase multi-enzyme complex (67, 68). ROS are important mediators of TB resistance (69–72) with functionality that includes direct mycobactericidal effects (73, 74) and remodelling of the host cell anti-*Mtb* response (75, 76). At 24 hours after infection, the transcriptomic responses of AM displayed IFN-γ signaling pathways in both phenotypic groups, with significantly stronger signaling in AM populations from LTBI samples (Figure 5E). This aligned with the observation of *Mtb-*triggered *IFNG* upregulation only in LTBI IEL-like L.3 cells and suggested a desensitization of resister AM to IFN-γ signaling, possibly due to constitutive exposure to high alveolar IFN-γ levels (77).

At 24 hours after infection, the stress-induced *MICA* gene was more strongly upregulated in resister AM in response to *Mtb* than LTBI AM. It is possible that this stronger *MICA* induction was a result of the stronger activation of the TNF signaling in resister AM, as TNF is capable of inducing MICA via NF-κB (78). As the main ligand of the NKG2D receptor, the presence of MICA in the membrane renders stressed and infected cells susceptible to killing by NKG2D expressed by cytotoxic cells (79). A contribution of the MICA/NKG2D axis to the resister trait is supported by the finding that cell-surface MICA is induced by *Mtb* infection, which leads to the killing of *Mtb*-infected DCs by NKG2D-expressing cytotoxic cells (80). The combined stronger induction of *MICA* in AM with the higher number of NKG2D-expressing poly-CTLs in resister alveoli provided a strong case that cytotoxic mechanisms are a main effector of increased resistance to infection with *Mtb*.

**Figure 7:**
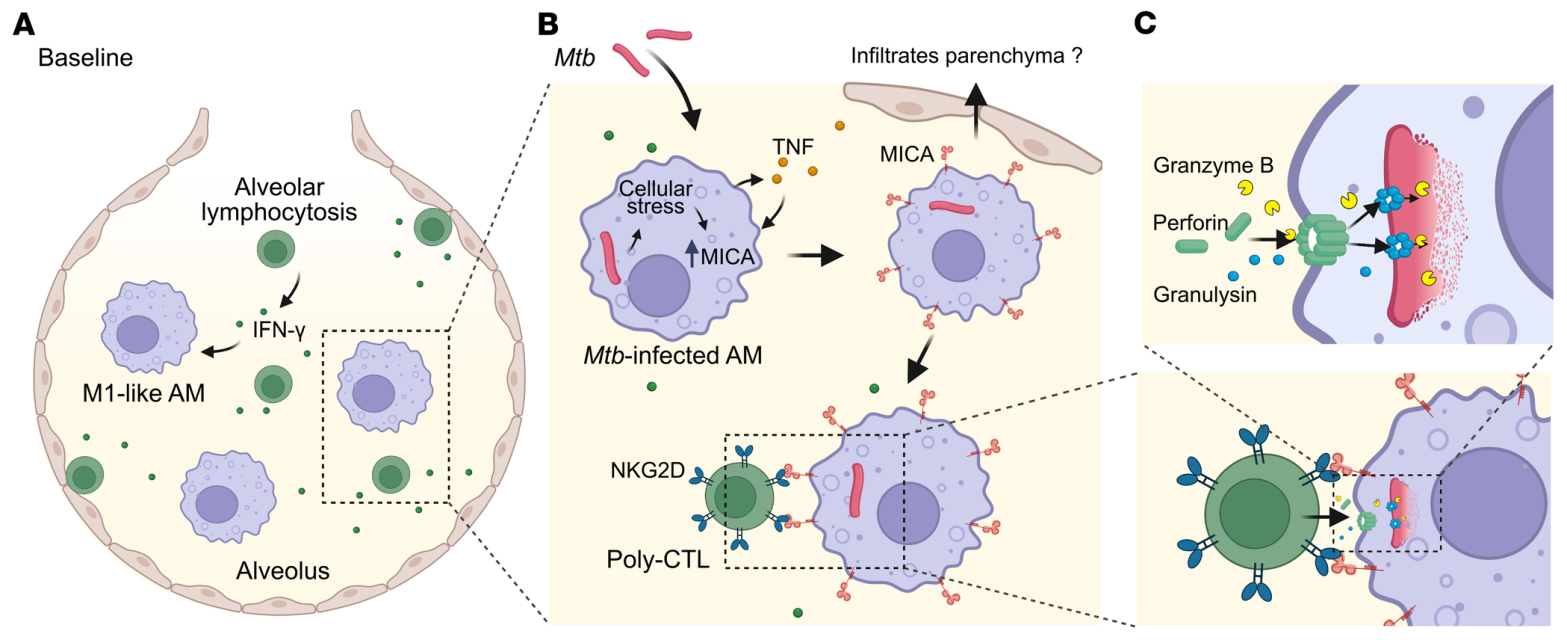
Proposed model for *Mtb* infection resistance in the sample of PWH investigated in our study. (**A**) At baseline, resisters display alveolar lymphocytosis resulting in increased constitutive levels of IFN-γ which pushes resister AM toward an M1-like state. (**B**) The AM preactivated state leads to increased TNF signalling, cellular stress, and upregulation of MICA after infection with *Mtb*. MICA is the ligand recognized by the activating NKG2D receptor expressed by poly-CTLs. (**C**) Poly-CTL coexpressing granzyme B, granulysin, and perforin are present in resister alveoli at more than 26 times higher numbers, leading to improved killing of infected AM and intracellular *Mtb* in alveoli of resisters. Created with BioRender.com. Poly-CTL, poly-cytotoxic (GNLY/GZMB/PRF1^+^) T lymphocytes (CD8^+^ T and γδ T).

Based on our results obtained by scRNA-Seq, we are proposing the following model to explain the resister phenotype in our sample of PWH (Figure 7). In the absence of *Mtb*, constitutive *IFNG* transcription in T cells combined with alveolar lymphocytosis results in increased levels of IFN-γ in resisters’ alveoli (Figure 7A). The increased level of IFN-γ pushes resister AM toward an M1-like physiological state. Infection with *Mtb* triggers in resister AM a higher production of TNF and significantly stronger TNF signaling relative to LTBI AM (Figure 7B). TNF signaling contributes to a strong upregulation of *MICA* transcription and presumably increased surface expression of the corresponding protein. MICA functions as the primary ligand for the NKG2D receptor (encoded by *KLRK1*), which in our sample is primarily expressed by PRF1^+^GZMB^+^GNLY^+^ poly-CTLs (Figure 7B). Both in the presence and absence of *Mtb*, *KLRK1* is expressed at 3-fold higher levels by resisters’ poly-CTLs. Since these cells are on average found in resisters at 26 times the number of those in LTBI alveoli, this represents a nearly 80-fold higher number of effective mycobactericidal poly-CTLs in resister versus LTBI alveoli. Poly-CTLs will kill infected cells through the action of perforin, which provides cytosolic access for granulysin and granzyme, 2 molecules that jointly lyse and kill *Mtb* (Figure 7C). Extension of this model from the alveolar resister concept includes the possibility that AM expressing an increased number of MICA ligands may escape into the lung parenchyma where they may seed the formation of protective granuloma (Figure 7B). Future functional validation of this hypothetical model would identify poly-CTLs as a prime target for a transmission-blocking TB vaccine.

## Methods

### Sex as a biological variable.

Sex was self-reported by the participants (1 male and 13 females, Table 1). No analysis by sex was performed due to the presence of only 1 male participant. This sex distribution reflects the sex distribution in the study population (12). Hence, sex-related effect cannot be captured in this study.

### Study participants and bronchoscopy.

The participants of this study are part of the ResisTB cohort, described in detail by Kroon et al. (12) and Gutierrez et al. (22). All participants enrolled in the ResisTB study are PWH with no history of TB while living in Cape Town, South Africa, an area of high *Mtb* transmission. The resister group, previously coined HITTIN (HIV-1 infected persistently TB, tuberculin, and IGRA negative), is composed of subjects with 3 consecutive IGRA negative assays and a TST equal to 0 mm. The LTBI group, previously coined HIT (HIV-1-infected IGRA positive, tuberculin positive) is composed of subjects with IGRA positivity in 2 consecutive tests and TST of 10 mm or more (Table 1). All participants tested positive for *Mtb*-specific antibodies, providing strong evidence that all had been exposed to *Mtb* (12). Since immune conversion was the key phenotype defining the groups of resisters and LTBI participants, we selected conservative cut-off values for group assignments (Table 1). All participants had a history of low peripheral CD4^+^ T cell counts (<200 cells/mm^3^), which were reconstituted after antiretroviral therapy (>400 cells/mm^3^) (inclusion criteria). For the present study, 14 participants (7 resisters and 7 LTBI) underwent a BAL procedure. Both groups presented similar peripheral nadir CD4 counts (average ± SD of 116 ± 59 cells/μL in resisters and 121 ± 29 cells/μL in LTBI, *P* = 0.805) and peripheral CD4 counts at the time of BAL collection (574 ± 150 cells/μL in resisters and 618 ± 128 cells/μL in LTBI, *P* = 0.639). Except for one LTBI participant, all participants were female. Participants self-identified as Xhosa, a major ethnic group in South Africa, except 2 individuals who self-identified as Sotho. The age and the years from HIV diagnosis/ART initiation (length of HIV/ART) were not significantly different between resister and LTBI participants. At the time of BAL fluid collection, the mean (± SD) age and HIV/ART length were respectively 49 ± 6 years and 12 ± 3 years in the resister and 49 ± 5 years and 13 ± 2 years in the LTBI group (Table 1). Still, to avoid residual confounding, all genetic analyses were adjusted on length of HIV/ART. All subjects were nonsmokers. Bronchoscopies with BAL were performed according to current recommendations (81, 82) in a research bronchoscopy facility (SU-IRG Biomedical Research Unit, Stellenbosch University [SU]) as recently described (42). In brief, all participants were prescreened for fitness for bronchoscopy according to predefined criteria by a study clinician with knowledge of the procedure. Individuals were excluded from this study if they presented with a cough of any duration, fever, or used antibiotics in the past 4 weeks before the bronchoscopy. Prior to the bronchoscopy, chest x-ray and TB sputum testing were performed. No lung parenchymal abnormalities were observed, and all participants tested negative for TB by sputum GeneXpert Ultra and liquid culture. No *Mtb* cultures or PCR for *Mtb* or respiratory viruses were performed on BAL samples. Bronchoscopies were performed under conscious sedation. The bronchoscope was targeted to lung regions affording ease of accessibility and the lavage was performed by instilling sterile saline solution at 37°C up to a maximum volume of 240 mL in aliquots of 60 mL at a time, with aspiration between aliquots. Aspirated fluid was collected in sterile 50 mL polypropylene tubes and transported on ice to the laboratory. BAL cell collection, culture, and infection with *Mtb* strain H37Rv were done as presented in the Supplemental Methods.

### ScRNA-seq and CITE-seq.

Single-cell capture and library preparation were performed with Chromium Next GEM Single Cell 3′ Reagents Kit, version 3.1 (10X Genomics, USA), as described in the Supplemental Methods. Cellular indexing of transcriptomes and epitopes by sequencing (CITE-Seq) for cell-surface markers was done using a TotalSeq-B Human TBNK cocktail of monocyte-, T-, B-, NK, and NKT-cell specific markers (BioLegend), as presented in the Supplemental Methods. Libraries were paired-end sequenced on Illumina NovaSeq 6000 S4 flowcells. We successfully generated 2 CITE-Seq and 55 scRNA-Seq libraries, while 1 noninfected 6-hour scRNA-Seq library from an LTBI subject failed in the library preparation and was not sequenced (Supplemental Table 1).

### SC data processing.

Combining the 55 scRNA-Seq libraries (from the 6-hour and 24-hour *Mtb*-infected and noninfected samples) with the 2 CITE-Seq samples (scRNA-Seq plus cell-surface antibody capture), we generated 57 scRNA-Seq libraries from the 14 subjects (Supplemental Table 1). Cell Ranger software, version 7.0.1 (10X Genomics), was used for alignment to the GRCh38 human genome and generation of feature-barcode matrices per library. Data analysis was done using Seurat, version 4.3.0 (83). Preprocessing quality control and cleaning steps are presented in the Supplemental Methods. At this step, 4 libraries prepared from 1 resister participant were excluded due to the high proportion of dead cells (Supplemental Table 1). To integrate all libraries, normalization was done with SCTransform and integration with the RPCA method from Seurat, version 4.3.0 (83, 84), using the whole BAL data in the first step and the myeloid and lymphoid cells separately in the second step. Unsupervised clustering followed by manual cell-type annotation were done as detailed in the Supplemental Methods. In total, we obtained 257,671 high-quality cells.

### DE analysis.

To perform DE analyses, we created pseudobulk expression matrices and used linear models in Limma, version 3.54.2, and accounting for covariates as detailed in the Supplemental Methods (85, 86). First, we performed a DE analysis of the myeloid clusters between resisters and LTBI samples in the absence of *Mtb* (baseline resister versus LTBI analysis). For multiple test correction, we used the Benjamini-Hochberg FDR. Genes were considered differentially expressed when presenting absolute log_2_FC > 0.2 and FDR < 0.1. Second, for the DE analyses for the ex vivo *Mtb* challenge (*Mtb*-response analysis) from the 2 postinfection time points (6 hours and 24 hours), 6 contrast tests were performed per cluster. In 4 contrasts, we tested the DE of genes in response to the *Mtb* challenge by group. For that, we compared the expression of the noninfected versus the infected libraries by group and time point: LTBI (6 hours), resister (6 hours), LTBI (24 hours), and resister (24 hours). In addition, we performed 2 contrasts per cluster where we compared the *Mtb* responses between resisters and LTBI by time-point (interaction analysis): resister (6 hours) versus LTBI (6 hours) and resister (24 hours) versus LTBI (24 hours) (see interpretation in the Supplemental Methods). For multiple test correction based on the different contrasts of *Mtb* response per cluster, we used the StageR FDR (87). Genes were considered differentially expressed when presenting absolute log_2_FC > 0.2 and FDR < 0.2.

Due to the low number of lymphocytes in LTBI BAL fluid, we investigated gene expression at the level of the 19 lymphocyte clusters of selected candidate genes with Seurat FindMarkers function for pairwise comparisons based on the cell group and conditions (see contrasts in Supplemental Methods). Absolute log_2_FC > 0.2 and Benjamini-Hochberg FDR < 0.2 were used as thresholds.

### GSEA analysis of Hallmark pathways.

For the GSEA analyses, we used the R package fgsea, version 1.28.0 (88), and tested the enrichment of 50 gene sets from the Hallmark pathway gene set collection from MSigDB (89). For that, the total tested genes were ranked based on the log_2_FC × –log_10_(*P*-value) in descending order (Supplemental Methods). Gene sets with absolute normalized enrichment score (NES) > 1.5 and FDR < 0.05 were considered significant.

### TF activity score.

A univariate linear model (ULM) was used to test the TF activity per cell using decoupleR, version 2.8 (90), and the TF-gene interaction reference from CollecTRI (91) as described in the Supplemental Methods. A *t* test with Benjamini-Hochberg correction was then used to evaluate significant differences in mean TF activity per cluster between cells from the 2 groups. A Benjamini-Hochberg correction was applied to calculate the FDR for all tested TF and AM/DC clusters. TF displaying FDR < 0.01 and absolute difference of normalized TF score > 0.2 were considered significant.

### Statistics.

Comparison of the clinical features or cell population proportions and ratios between the LTBI and resister were done using unpaired 2-tailed *t* test or Wilcoxon’s tests with Bonferroni’s multiple test correction as indicated in the main text or figure legends. We used box plots to present the population proportions by group, where the band in the box plot indicates the median, the box indicates the first and third quartiles, and the whiskers indicate ± 1.5× interquartile range.

### Study approval.

Research was performed in accordance with the Declaration of Helsinki, and all participants provided written informed consent for the study procedures, which were approved by the SU Health Research Ethics Committee (N16/03/033), the SU Research Ethics for Biological and Environmental Safety Committee (BES-2023-19406), and the Research Institute of the McGill University Health Centre (MP-CUSM-15-406).

### Data availability.

The scRNA-Seq fastQ files and CellRanger feature-barcode matrices from the 6 resisters and 7 LTBI participants are deposited in the NCBI’s Gene Expression Omnibus database (GEO GSE273373). No original code is reported. Values for all data points in graphs are reported in the Supporting Data Values file.

## Author contributions

MDS, VMF, MO, AC, EGH, LA, MM, JLC, GW, NDP, and ES performed study design and conceptualization. MDS performed the scRNA-Seq data processing and analysis. VMF performed data analysis for TF and cell-cell communication. STM and CEM performed clinical procedures. STM, CEM, EEK, and MM recruited and enrolled subjects. MO performed the cell-based experiments for the scRNA-Seq. MM and ES performed project administration. GW and NDP performed data collection. EGH, NDP and ES produced funding sources. ES supervised the project. MDS, VMF, MO, AC, SBD, EGH, LA, MM, JLC, GW, NDP, and ES contributed to the data interpretation. MDS and ES wrote the draft of the manuscript and VMF, STM, MO, AC, SBD, LA, MM, JLC, GW, and NDP reviewed and edited it. MDS and VMF worked on visualization. All authors read the final manuscript.

## Supplementary Material

Supplemental data

ICMJE disclosure forms

Supplemental tables 1-11

Supporting data values

## Figures and Tables

**Table 1:**
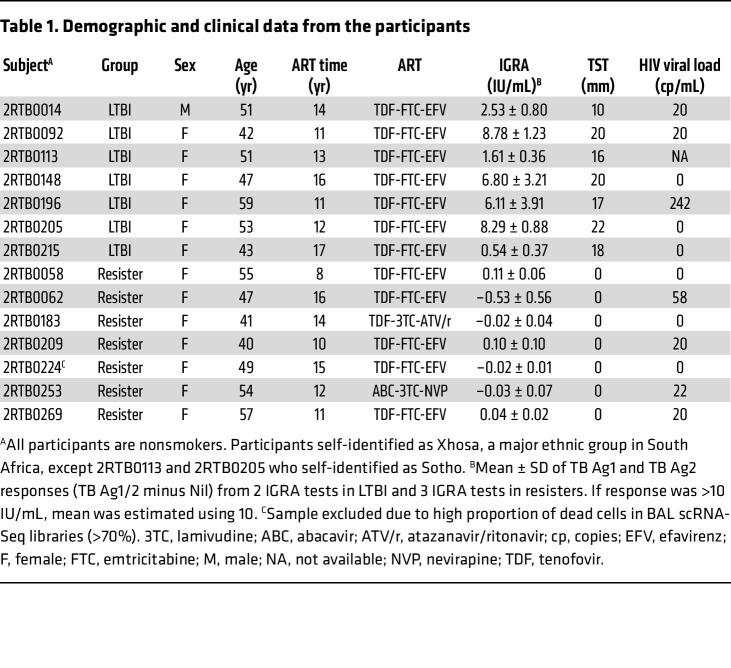
Demographic and clinical data from the participants

## Version 1 01/21/2025

In-Press Preview

## Version 2 04/01/2025

Electronic publication
