# Supplementary material for: Mycobacterium tuberculosis resisters despite HIV exhibit activated T cells and macrophages in their pulmonary alveoli
Source: J Clin Invest. 2025 Jan 21;135(7):e188016. doi: 10.1172/JCI188016 (PMC11957701; doi:10.1172/JCI188016)
Supplement: Supplemental data [file jci-135-188016-s185.pdf]

Supplemental figures

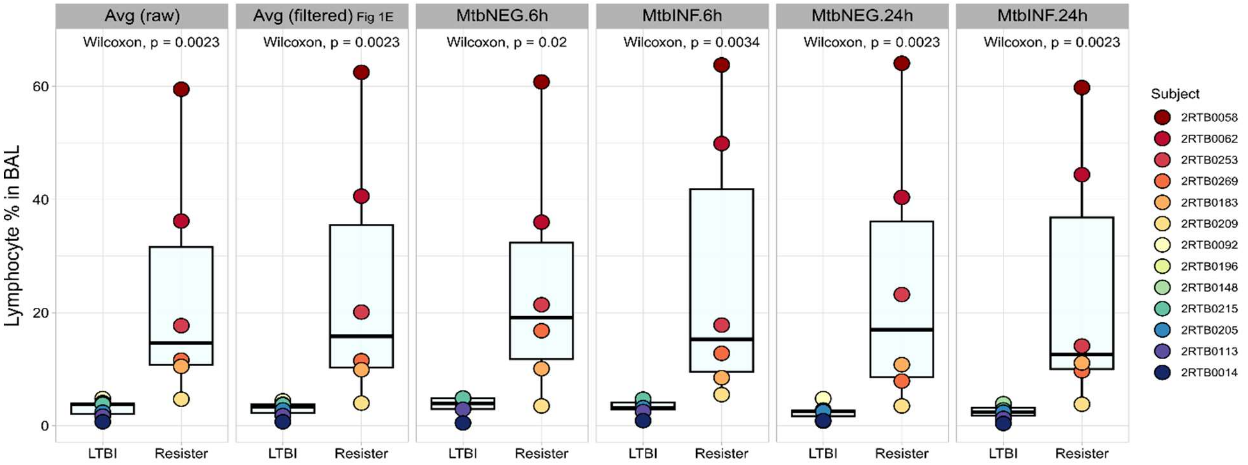

Supplemental Figure 1. Consistent lymphocyte proportions among scRNA-seq libraries from the same BAL samples. Box plot of lymphocyte proportion in BAL samples obtained from resister and LTBI participants. Each dot and its colour represent the cells collected from each subject. First and second panels present the percents per subject (cells from all libraires combined), calculated from the raw data (dead and live cells) and filtered data (after pre-processing quality control cleaning), respectively (Supplemental Table 1). Third to sixth panels present the percent of lymphocytes by library in non-infected 6h (MtbNEG.6h), *Mtb*-infected 6h (MtbINF.6h), non-infected 24h (MtbNEG.24h) and *Mtb*-infected 24h (MtbINF.24h) libraries, respectively.

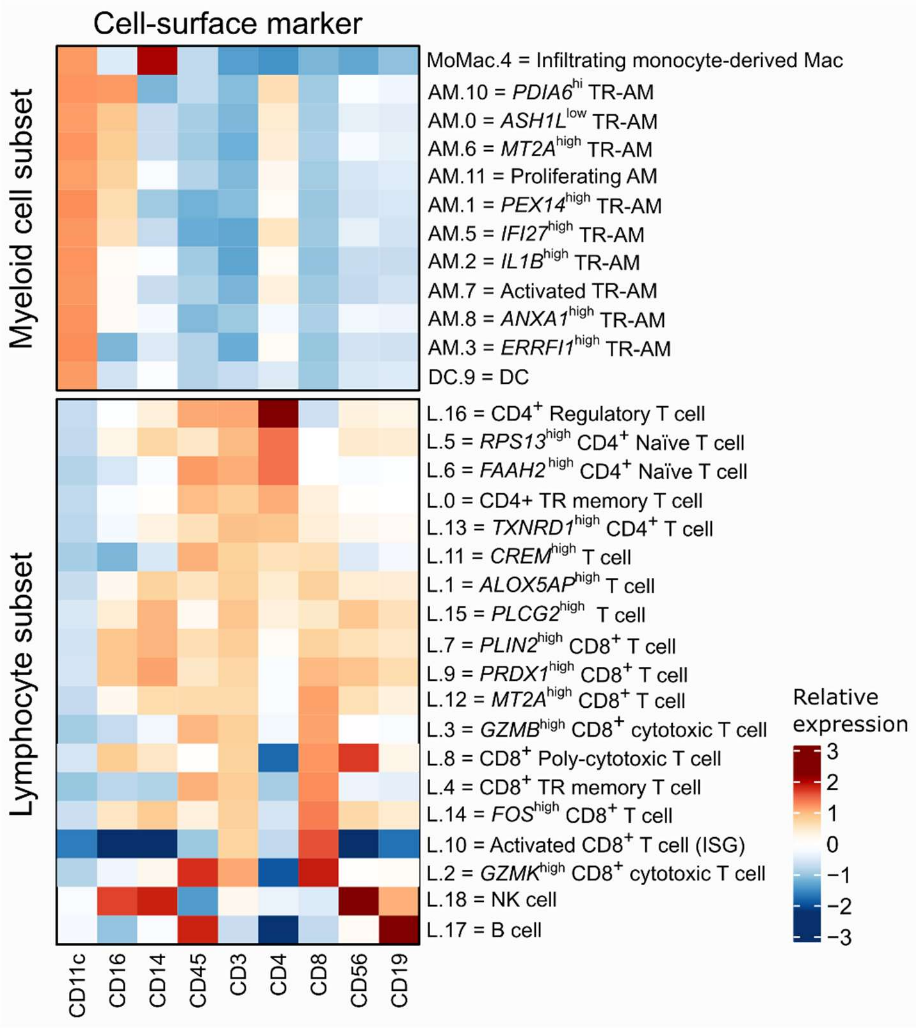

Supplemental Figure 2. Heatmap representation of the normalized expression of cell-surface markers in each myeloid cell and lymphocyte subpopulation. In the plot, AM clusters are ordered by their CD14 and CD16 expressions and T cell clusters by their CD4 and CD8 expressions. The colour scale indicates the relative expression (Z-score). Cluster annotations are shown on the right of the graph. Data from two non-infected samples with approximately 2h of manipulation. AM: alveolar macrophage, DC: dendritic cell, ISG: interferon-stimulated genes, Mac: macrophage, NK: natural killer, TR: tissue-resident.

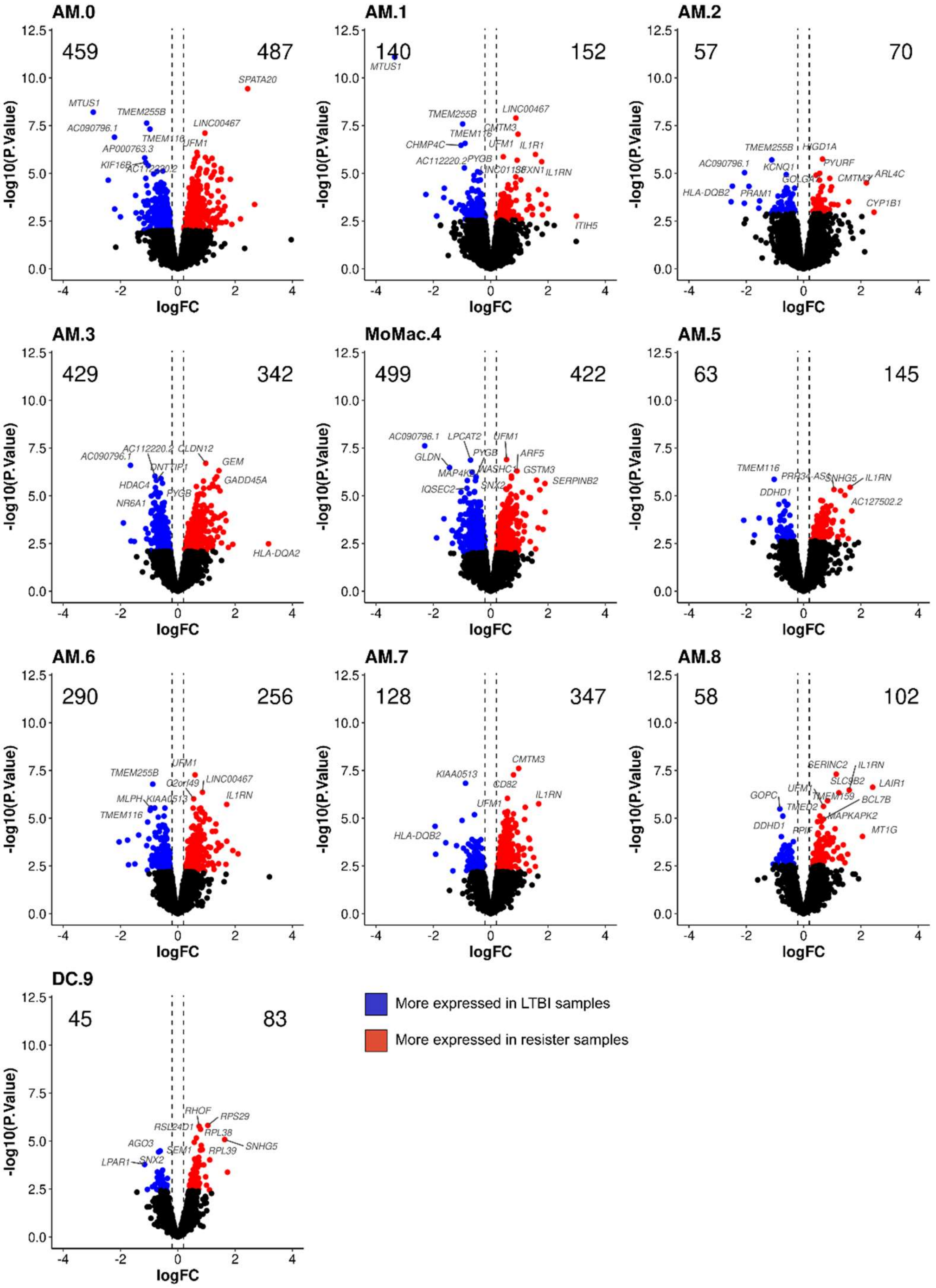

Supplemental Figure 3. Differential expression analysis in the absence of *Mtb* of myeloid cell subpopulations from resister vs LTBI samples. Volcano plots show the results from the pseudobulk DE analysis by myeloid cell subpopulation. The x-axes show the log2 fold-change (log2FC) difference of gene expression between resister and LTBI samples in the absence of *Mtb* per cluster. The y-axes present the -log10 of the unadjusted *P*-value for significance of expression differences. Dashed lines correspond to the log2FC thresholds of -0.2 and 0.2. DEG with higher expression in cells from resisters or LTBI are shown in red and blue, respectively (FDR < 0.1). Total numbers of DEG higher or lower expressed in resister samples are indicated in the top corners.

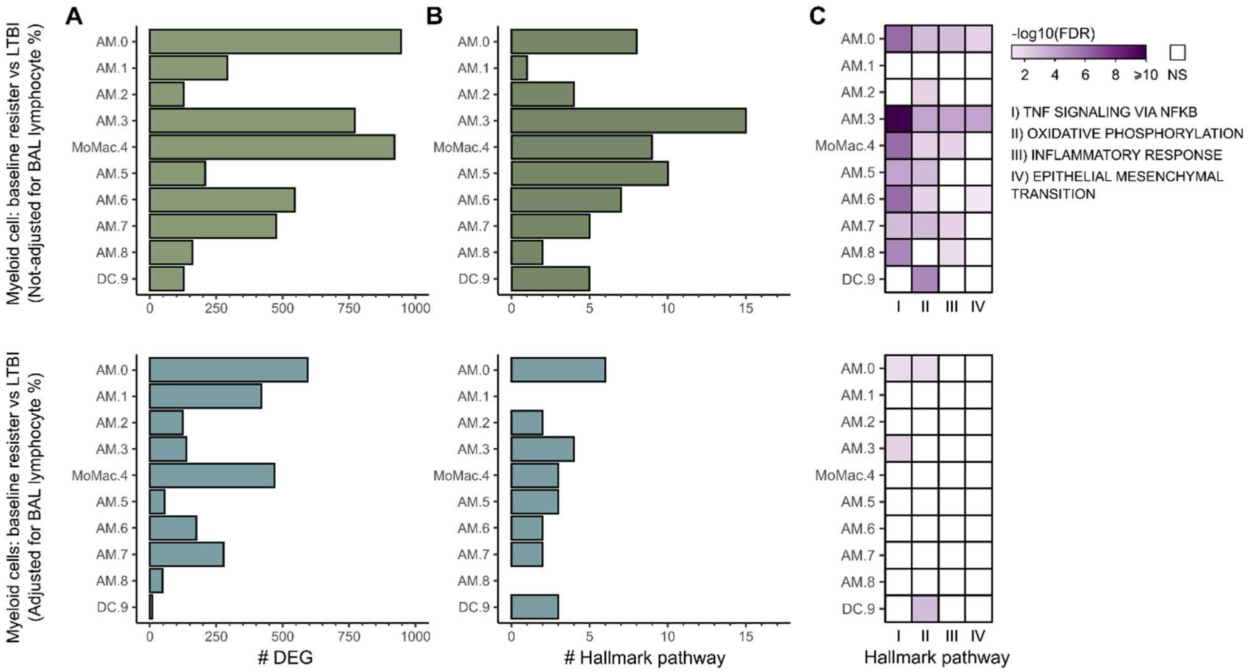

Supplemental Figure 4. Impact of adjusting on the lymphocyte proportion in the baseline comparative analysis of resister with LTBI myeloid BAL cells. (A-C) Comparison of the results from the baseline analysis between myeloid cell subpopulations from resister and LTBI BAL samples without (top) and with (bottom) regressing-out the BAL lymphocyte proportion in the model. (**A)** Bar plots comparing the number of differentially expressed genes (DEG) between the two tests. (**B)** Bar plots comparing the number of significant Hallmark pathways between the two tests. **(C)** Heatmaps of selected Hallmark pathways enriched for genes with higher expression in resister compared to LTBI in the absence of *Mtb* without and with the adjustment on BAL lymphocyte proportion. The colour scale corresponds to the -log10(FDR). Non-significant (NS) results (FDR > 0.05) are shown in white.

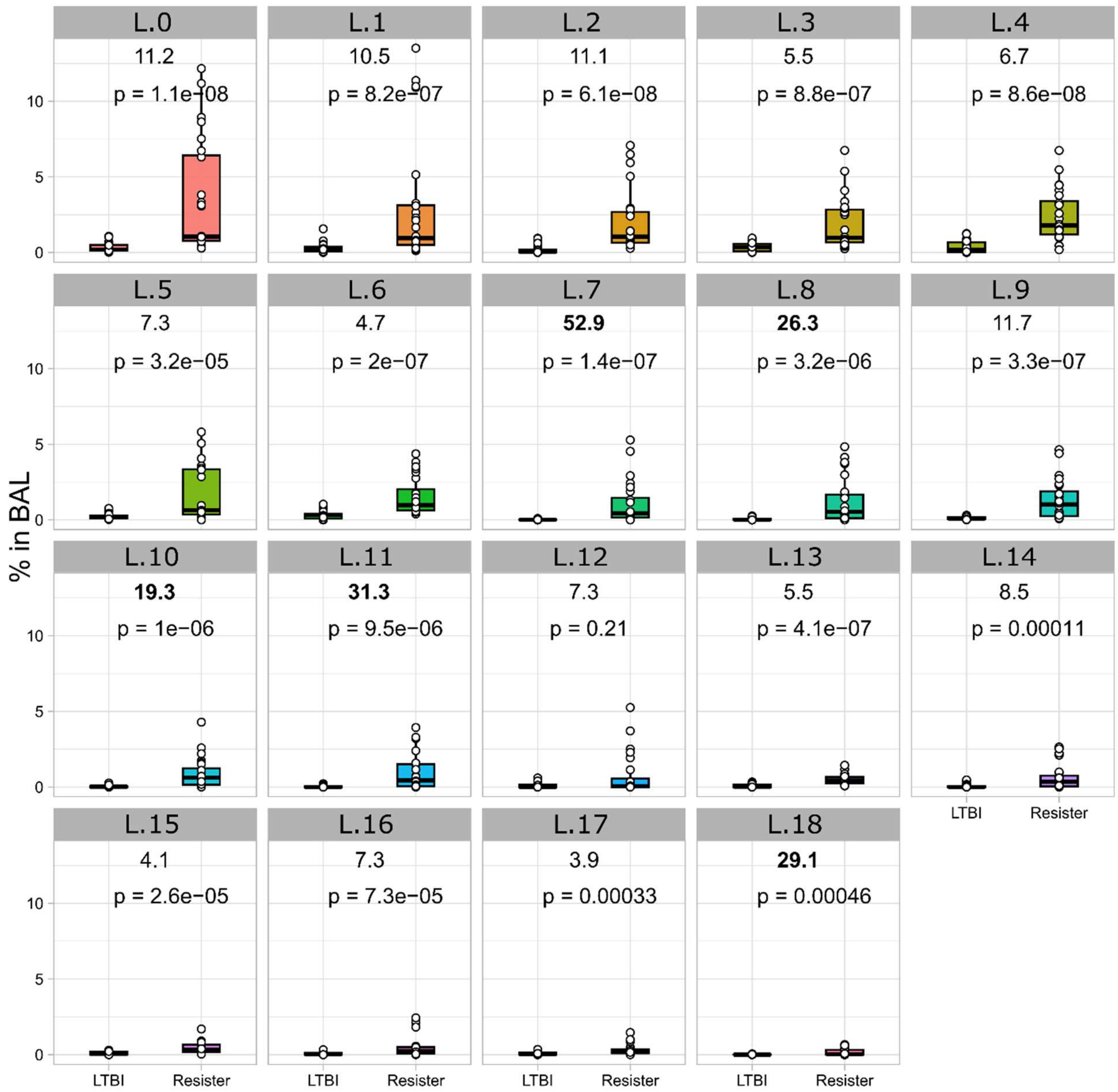

Supplemental Figure 5. Box plot of lymphocyte cluster proportions relative to total BAL cells from resister and LTBI samples. Each graph shows the cluster proportion where each dot represents the estimate obtained from one library. Boxes are coloured based on the clusters. Fold-enrichments from resister samples compared to LTBI (mean % resister/ mean % LTBI) are shown on top for each graph. The top 5 highest fold-enrichment are shown in bold. The range of mean % was 0.006-0.36% in LTBI and 0.18-3.51% in resisters. Of note, enrichment scores do not well represent cluster contribution to lymphocytosis as they are independent of the cluster sizes. For example, L.7 (enrichment score 52.9x) represents mean of 1.069% of BAL cells in resisters vs 0.02% in LTBI while L.0 (enrichment score 11.2x) represents 3.51% in resister vs 0.312% in LTBI. *P*-values obtained from a two-sided Wilcoxon test are shown below the fold-enrichments. Nominal *P*-values lower than $P < 0.0026$ passed the Bonferroni multiple test correction threshold (0.05/19). Library data from all conditions.

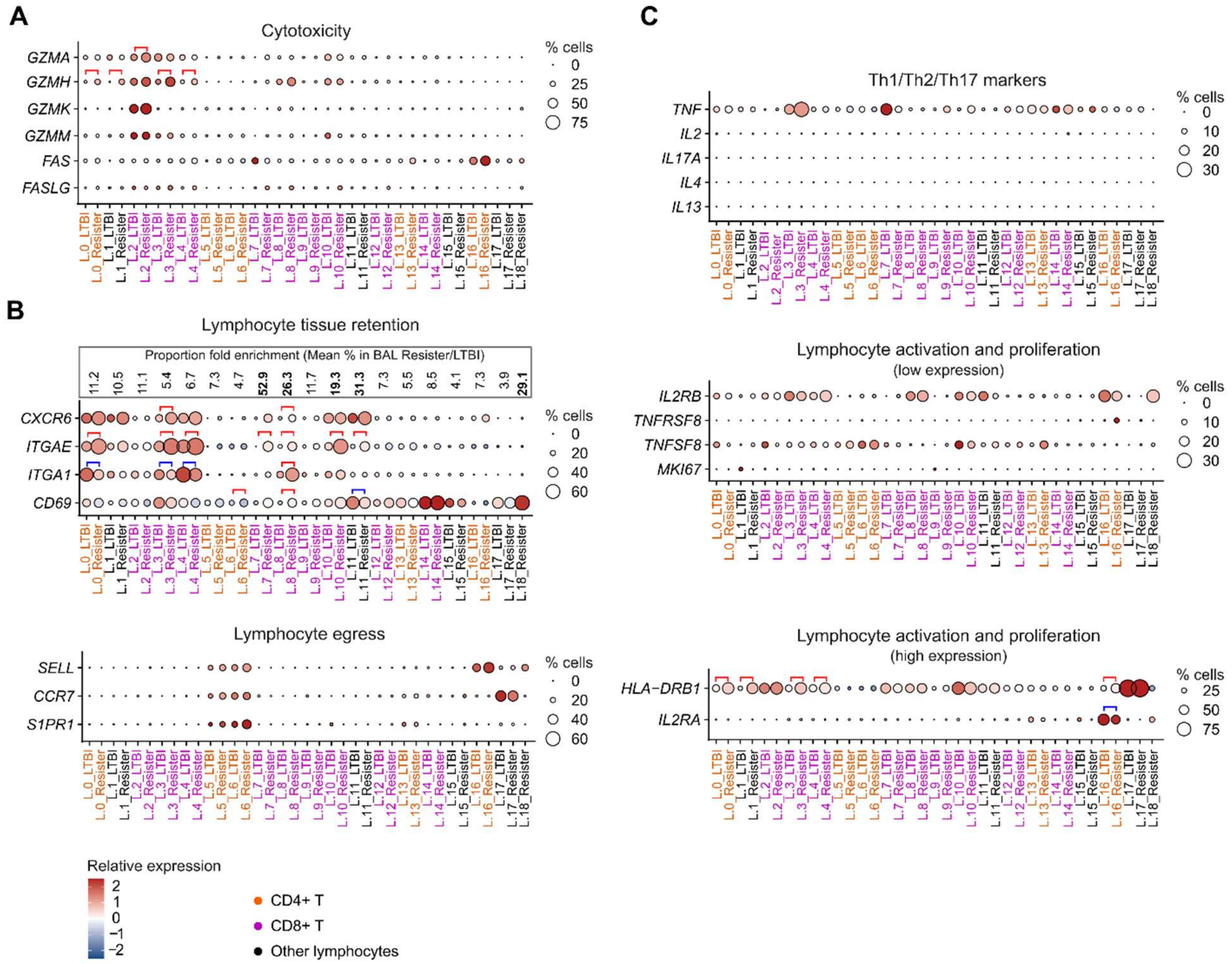

Supplemental Figure 6. Gene expression of selected genes in the 6h non-infected lymphocyte subpopulations from resister and LTBI BAL. Selected genes are involved in or are markers of **A)** lymphocyte cytotoxicity, **B**) lymphocyte tissue retention and egress, and **C**) Th1/Th2/Th17 cells and lymphocyte proliferation and activation. Lymphocyte clusters are shown on the x-axis and are separated by group (data from 6h non-infected cells). Cluster colours indicate the main cell-types. Colour and size of the circles correspond to the scaled expression and the percentage of cells expressing the gene by cluster, respectively. Red and blue lines indicate the genes expressed in >25% of the cells in at least one group which presented higher expression in resister or LTBI cells, respectively (absolute Log2FC > 0.2 and FDR < 0.2). Additional genes involved in activation (*IFNG*) and cytotoxicity (*GNLY, GZMB* and *PRF1*) are shown in Figure 4E and Figure 4F, respectively. In **(B)**, the fold enrichment of the cluster proportion in resister BAL relative to LTBI (Supplemental Figure 5) is shown on top to compare the BAL % fold enrichment and the expression of the tissue-retention markers. There were higher baseline expression levels of the *GZMA*, *GNLY* and *GZMH* (cytotoxicity, panel "A" and Figure 4F), *CXCR6* and *ITGAE* (tissue retention, panel "B") and *HLA-DRB1* and *IFNG* (cell activation, panel "C" and Figure 4E) in one or more resister lymphocyte clusters compared to LTBI. *ITGA1* presented higher or lower levels in resister cells depending on the cluster (tissue retention and T activation, panel "**B**"). *IL2RA* had lower expression in resister Treg (panel "C").

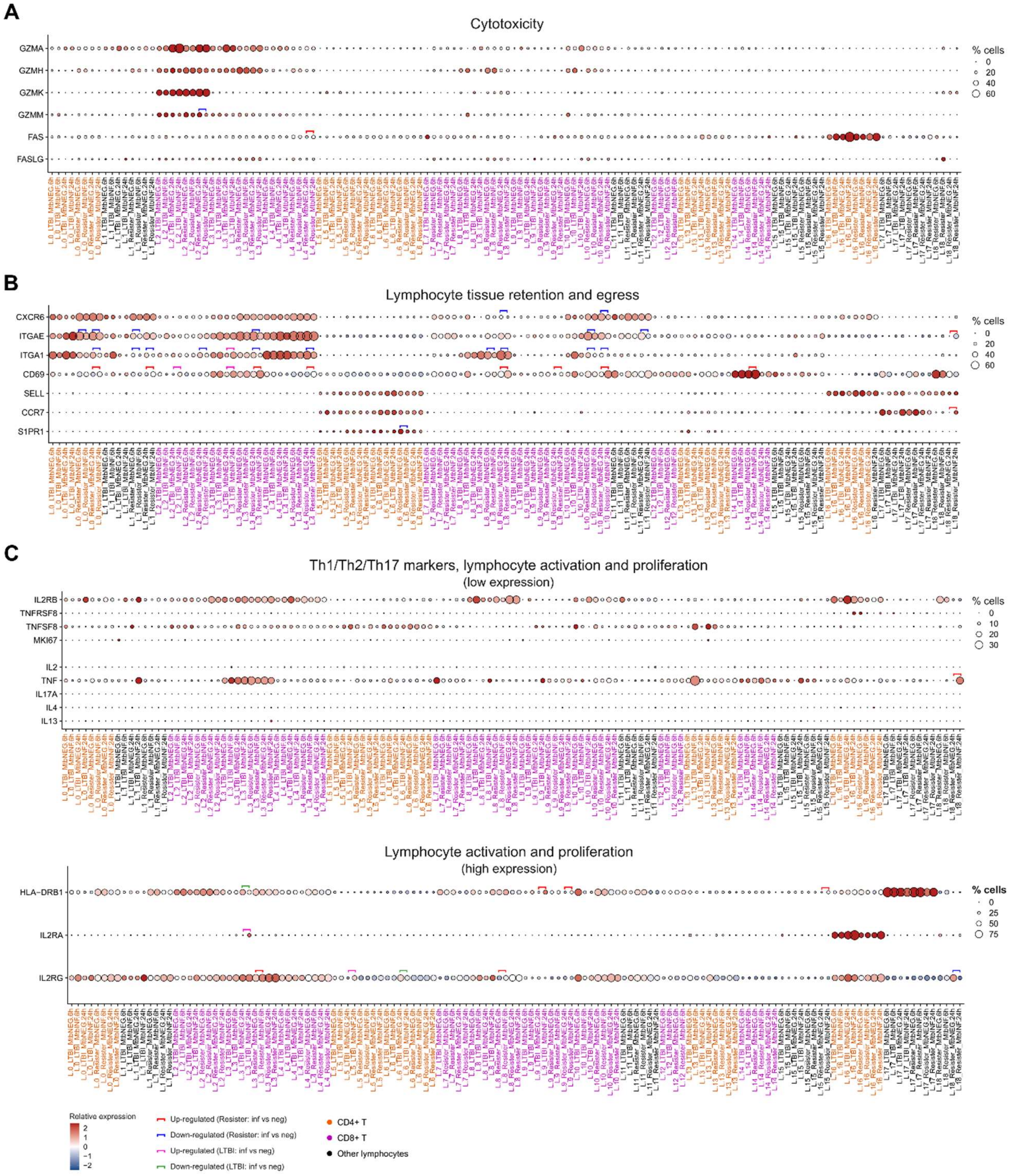

Supplemental Figure 7. Gene expression of selected genes in the *Mtb*-challenged and non-infected lymphocyte subpopulations from resister and LTBI BAL at 6h and 24h p.i. Selected genes are involved in or markers of **A**) lymphocyte cytotoxicity, **B**) lymphocyte tissue retention and egress, and **C**) Th1/Th2/Th17 cells and lymphocyte proliferation and activation. Lymphocyte clusters are shown on the

x-axis and are separated by group, time-point and infection status as indicated in the name. Cluster colours indicate the main cell-types. Colour and size of the circles correspond to the scaled expression and the percentage of cells expressing the gene relative to the total number of cells in the cluster (in the specified group and condition), respectively. Coloured lines indicate the DEG genes in the contrast of infected vs non-infected cells by group and time-point (expressed in >25% of the cells in the infected and/or non-infected & absolute Log2FC > 0.2 & FDR < 0.2). DE results in Supplemental Table 11. Additional genes involved in activation (*IFNG*) and cytotoxicity (*GNLY* and *GZMB* and *PRF1*) are shown in Figure 6A and Figure 6B, respectively. MtbNEG: Non-infected cells. MtbINF: *Mtb*-challenged samples (bystander + infected cells). Mostly in 24h *Mtb*-response in the resister cells, there was a reduction of *ITGAE* and *ITGA1* expressions (integrins involved in cell-cell adhesion and tissue retention) and an induction of *CD69* expression (a tissue residency and T activation marker) in several T clusters (panel "B").

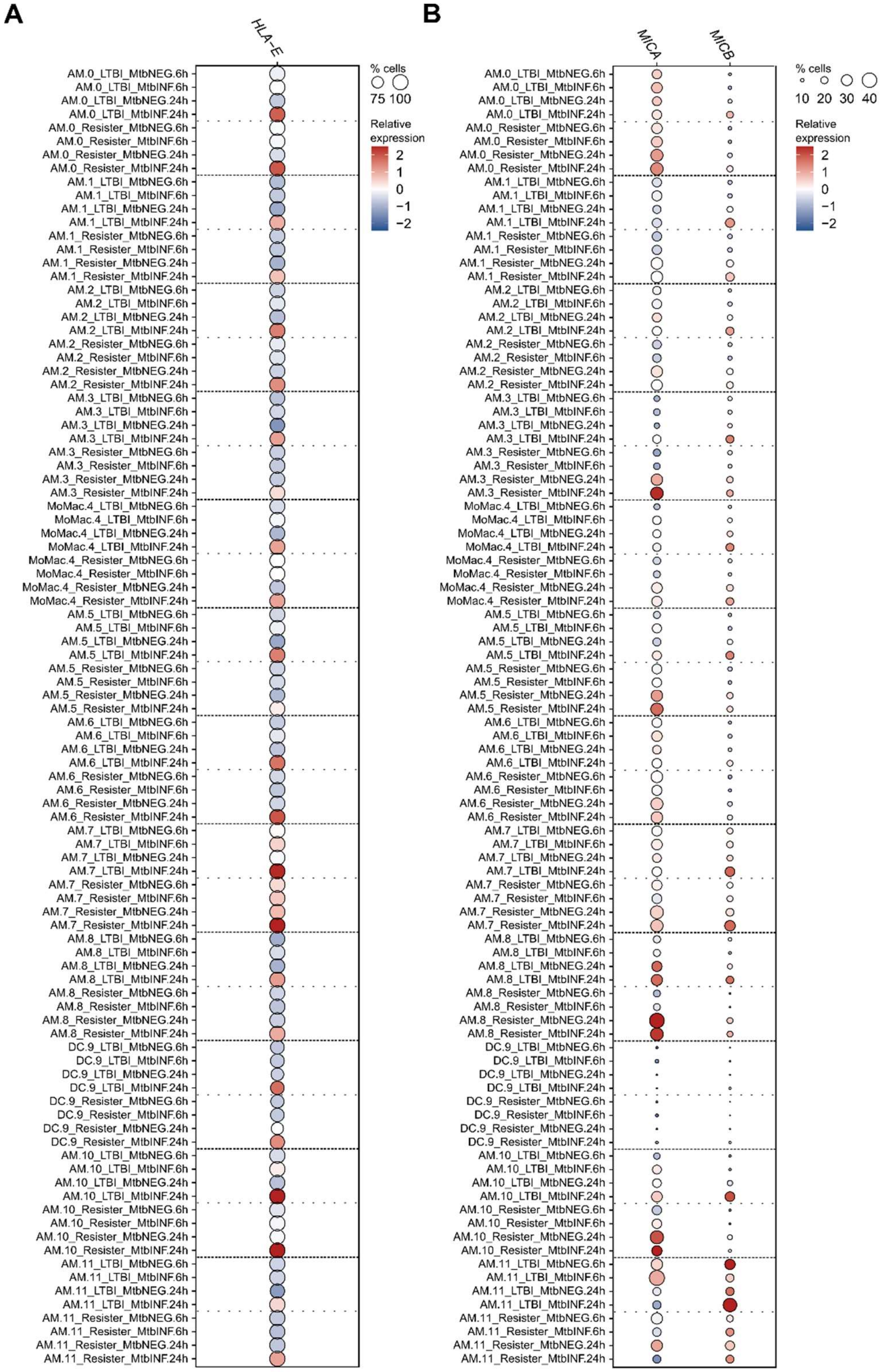

Supplemental Figure 8. *HLA-E*, *MICA* and *MICB* expressions at 6h and 24h of *in-vitro* culture in presence and absence of *Mtb* in LTBI and resister myeloid cell subpopulations. (**A**) *HLA-E* and (**B**) *MICA* and *MICB* expression in the myeloid cells by group and condition. For each cluster, the size of the circles indicates the proportion of cells expressing the gene. Circle colours indicate the scaled expression in the proportion of positive cells relative to all the clusters and conditions (scaled by gene).

Supplemental methods

Blood count

Blood was collected by phlebotomy in a heparinized vacutainers and PBMC isolated according to the standard Ficoll isolation method. PBMC were cryopreserved in 10% dimethyl sulfoxide (MilliporeSigma, Massachusetts, USA) and 90% fetal bovine serum (Cytiva, Massachusetts, USA). Differential counts were performed on PBMC by standard flow cytometry staining for the markers (CD45, CD3, CD4, CD8, CD19 and CD14).

BAL cells collection

BAL fluid was placed on ice immediately after aspiration. Processing was initiated within two hours of collection. If the pellet was judged contaminated with blood by visual inspection, an additional red cell lysis step was performed in 1 ml of Lonza ACK lysis buffer (1x) [Whitehead Scientific (Pty) Ltd, SA]. The total cell count was conducted using a haemocytometer and viability check by Trypan Blue exclusion method. A fraction was used for a differential count by cytospin (Simport, Saint-Mathieu-de-Beloeil, Canada). BAL cells were cryopreserved in 10% dimethyl sulfoxide (MilliporeSigma, Massachusetts, USA) and 90% fetal bovine serum (Cytiva, Massachusetts, USA) and, by gradual cooling to -80 °C in a Nalgene Mr Frosty™ container [Sigma Aldrich (Pty) Ltd, Gauteng, South Africa] with isopropanol for 24h followed by long term preservation in liquid nitrogen.

BAL cell culture

Cryopreserved BAL cells were set in culture following a dropwise thawing protocol as follows. The content of a cryovial was thawed at 37°C in a water bath for 2 minutes. The cell suspension was transferred into a sterile 50ml tube. Dropwise, 1ml of RPMI-1640 with L-glutamine (Gibco, USA) with 50% heat-inactivated fetal bovine serum (hiFBS) (Gibco, USA), 1% penicillin/streptomycin (Gibco, USA), 10mM HEPES (Gibco, USA), 1% non-essential amino acids (Gibco, USA) and 5µg/ml Amphotericin B (Gibco, USA), 20µg/ml DNAse I (Roche, Germany) was added to the cells reaching a total volume of 32ml. The tube was spun at 320g for 12 minutes at room temperature. Cell pellet was dislodged and resuspended for the second wash in 20ml of RPMI-1640 containing 20% hiFBS, 10mM HEPES (Gibco, USA), 1% non-essential amino acids (Gibco, USA) and 2.5µg/ml Amphotericin B (Gibco, USA), 20µg/ml DNAse I (Roche, Germany). Cell were seeded at 2.5×10^5 cells per well in 24 well-plates Nunclon Sphera Low-attachment (ThermoFisher, USA) in 500µl of culture medium (RPMI-1640 with L-glutamine (Gibco, USA), containing 10% human serum (heat-inactivated AB+ off the clot, Wisent, Canada), 10mM HEPES (Gibco, USA), 1% non-essential amino acids (Gibco, USA) and 2.5ng/ml Amphotericin B (Wisent, Canada) and incubated at 37°C, 5% CO_2, and 95% relative humidity.

Mycobacterial cultures and BAL cell *Mtb* infection

Virulent *Mtb,* strain H37Rv, was grown in a liquid culture of Middlebrook 7H9 medium (BD Difco, USA) containing 0.2% glycerol (Fisher, USA), 0.05% Tween-80 (Sigma-Aldrich, USA) and 10% albumin-dextrose-catalase (BD, USA) at 37°C in rolling incubators. Bacteria were

grown to log phase determined by an optical density of 0.6 to 0.8 at 600nm, prior to inoculum preparation. Further, bacterial cultures were spun for 15 minutes at 3700 rpm, resuspended in RPMI-1640 and dislodged with a 22G needle. Cell suspensions were filtered through 5µm filters (Millipore, USA) to ensure single mycobacteria suspensions for BAL cell challenges. Bacterial counts of inocula were done using disposable Neubauer hemocytometer (C-Chip, INCYTO, South Korea). Bacterial loads were confirmed by colony-forming unit (CFU) counts by plating serial dilution of inoculum in 7H9 growth medium on Middlebrook 7H10 agar (BD, USA) plates containing 0.5% glycerol and 10% oleic acid-albumin-dextrose-catalase (BD, USA). Colonies were counted 4 weeks post-plating. BAL cells were infected on average at a multiplicity of infection (MOI) of 6.5:1 for 6h and 24h at 37°C, 5% CO_2, and 95% relative humidity. In parallel, non-infected samples were incubated for the same periods.

Single cell RNA library preparation and sequencing

After incubation, BAL cells were collected and washed once in cold PBS (Wisent, Canada) containing 1% bovine serum albumin (Wisent, Canada). Cell clumps were removed by passing cell suspension through 40µm FlowMi strainer (Bel-Art, USA). Single cell capture and library preparation was performed with Chromium Next GEM Single Cell 3′ Reagents Kit v3.1 (10X Genomics, USA). Cell suspensions were loaded on a Chromium Next GEM Chip G (10X Genomics, USA) together with gel beads from Chromium Next GEM Single Cell 3′ GEM Kit v3.1 and captured on Chromium Controller (10X Genomics, USA) with recovery target of 1×10^4 cells. cDNAs were generated following the 10X Genomics protocol CG000315 and their quality was checked with Bioanalyzer High Sensitivity DNA Kit (Agilent, USA). One quarter of the

total cDNA was used to generate sequencing libraries using Library Construction Kit (10X Genomics, USA) and barcoded using Dual Index plate TT set A (10X Genomics, USA). Obtained libraries were double side size-selected using SPRIselect beads (Beckman Coulter, USA) to enrich for fragments 300-800 base pairs long, centered at 450bp. Libraries were checked for quality with Bioanalyzer High Sensitivity DNA Kit and paired-end sequenced on Illumina NovaSeq 6000 S4 flowcells aiming to obtain 50,000 reads per cell. We aimed for generating 56 scRNA-seq libraries from the 14 participants, which included libraries from four conditions per subject based on the infection status and time of incubation of the cells: i) *Mtb*-infected 6h, ii) non-infected 6h, iii) *Mtb*-infected 24h and iv) non-infected 24h (Supplemental Table 1). We successfully generated 55 scRNA-seq libraries, while one non-infected 6h library from an LTBI subject failed in the library preparation and was not sequenced (Supplemental Table 1).

Surface markers staining for single-cell data analysis

To facilitate the characterization of BAL cell sub-populations of leukocytes, we performed CITE-seq (cellular indexing of transcriptomes and epitopes by sequencing) with non-infected cells from two participants (one LTBI and one resister), using a TotalSeq-B Human TBNK cocktail of monocyte-, T-, B-, NK, NKT-cell specific markers (BioLegend, USA). Following the 10X Genomics protocol CG000149_RevD, 1 µg of Antibody cocktail was used per 1×10^6 BAL cells in 100 µL staining volume. Using Chromium Controller, Chromium Next GEM chip G and Chromium Next GEM Single Cell 3′ Reagents Kit v3.1 (10X Genomics, USA) cell emulsions, with a target capture of 1×10^4 cells, were obtained for scRNA-seq and Cell Surface Protein

library preparations. Following the 10X Genomics protocol CG000317_RevD, cDNA and DNA from cell surface protein Feature Barcode were amplified using Feature cDNA Primers 2 from 3' Feature Barcode kit (10X Genomics, USA). After amplification step, samples were size-selected with SPRIselect beads (Beckman Coulter, USA), where cDNA bound to beads, while DNA from Cell Surface Protein Feature Barcode remained in the solution. After magnetic separation, cDNA was eluted from beads and used for scRNA-seq libraries as described above, whereas supernatants were used for Cell Surface Protein Library construction. The Cell Surface Protein Feature Barcode DNA was purified by an additional round of SPRIselect beads precipitation and amplified with primers from the Dual Index plate NT set A (10X Genomics, USA). Library quality was assessed with Bioanalyzer High Sensitivity DNA Kit and paired-end sequenced on Illumina NovaSeq 6000 S4 flowcells aiming to obtain 10,000 reads per cell.

SC preprocessing and data integration

Seurat objects for each library were created using CreateSeuratObject function with min.feature = 300, and gene expressions were normalized using "LogNormalize" method from Seurat NormalizeData function with default setting (1). An initial annotation of main cell-types in the raw data was done for quality control and filtering. Annotation was based on gene expression of canonical markers for tissue-resident alveolar macrophages (TR-AM; *CD68, MARCO, PPARG*), infiltrating monocyte-derived macrophages (MoMac; *CD68, CSF1R, CCL2*), dendritic cells (DC; *LAMP3, CCR7*), T/NK cells (*CD3D, TRAC, NKG7*), B cells (*CD79A, MS4A1*), neutrophils (*FCGR3B*) and erythrocytes (*HBB*). Neutrophils and erythrocytes totaled less than 50 cells in our whole dataset and were excluded from the analysis. We used Seurat AddModuleScore to search

for other known cell-types based on gene-sets from a previous study of human lung atlas (2). No additional cell-types were found in our BAL samples. We filtered low-quality cells and doublets based on the gene count per cell, where cells falling outside the interval of -1.5SD to +2SD were excluded (3). As the myeloid cells presented higher overall gene count per cell (~3k) compared to the lymphoid cells (~1k), the -1.5SD-+2SD gene count/cell filtering was done separately by main cell-type. Cells with more than 20% mitochondrial genes were also excluded as they were likely dead cells. Contaminated cells were excluded with DecontX (4), implemented in Celda v1.10.0 (5). Doublets were removed using DoubletFinder v2.0.3 using default parameters and manually curated based on co-expression of the canonical markers for the main cell-types (6). At this step, four libraries prepared from one resister participant were excluded due to the high proportion of dead cells (Supplemental Table 1). Next, we combined the remaining 53 libraries that passed the pre-processing filtering (Supplemental Table 1). To help the integration and clustering by increasing the sample size, we included 10 in-house scRNA-seq libraries prepared from fresh BAL (these samples were not included in the downstream analyses and otherwise are not part of the results shown in this study). To integrate all libraries, normalization was done with SCTransform and integration with the RPCA method from Seurat v4.3.0 (1, 7). This step was done with the top 1000 variable genes excluding mitochondrial and ribosomal genes (3). For visualization, UMAP was used as dimensional reduction method, using the top 25 PCs.

Cluster identification from the BAL cells

In the UMAP from all the BAL cells, we observed that the cells were separated into two main subsets of cells that were identified as lymphoid (T/NK/B cells) and myeloid (AM/MoMac/DC)

cells. To identify subpopulations of cells, the cells from the two main populations were separated based on the UMAP coordinates and each subset was re-integrated using the same method as the initial integration. Clustering was done with Seurat FindNeighbors and FindClusters functions. Parameters for the clustering were selected based on the cleanest separation found between T (*CD3D*$^{+}$) and NK (*CD3D*$^{-}$) cells in the lymphocyte subset and between TR-AM (*MARCO*^{high}) and MoMac/DC (*MARCO*^{low}) in the myeloid cell subset. Hence, clustering was done using the first 25 PCs and resolution of 1.2 for the lymphocyte subset and of 0.8 for the myeloid cell subset. An additional step of data cleaning was done to remove remaining low-quality cells and likely doublets of cells from the same main cell-type. For that, cells that were outliers in the UMAP for each cluster were removed. Three rounds of re-integration and cleaning were performed per subset. Libraries with less than 100 lymphocytes could not be included in the re-integration due to the low number of cells (Supplemental Table 1). For these libraries, we used Seurat FindTransferAnchors and TransferData functions to annotate the lymphocyte clusters, which was used for the cluster proportion estimates.

The cluster annotation was based on three analyses done in parallel: i) we compared the expression of known canonical markers among the different clusters, ii) we compared the expression of the cell-surface markers from the two CITE-seq samples and iii) we performed a DE analysis to compare the gene expressions among clusters. To identify the DEG among clusters in the single cell data, we used Wilcoxon test as implemented by Seurat FindConservedMarkers function. The DE analysis was done between the cells from a cluster against all the remaining cells from the main population. This was done combining the cells from the two groups and incubation time-points but separated by the infection status. Genes were considered differentially expressed if presenting FDR < 0.05, expression in $> 25\%$ of the cells

and absolute log2FC > 0.25 between the cluster and remaining cells in both tests: in the *Mtb*-infected and in the non-infected cells.

Comparison of the cell population proportions and the CD4/CD8 T cell ratio between the LTBI and resister from BAL and PBMC samples were done using two-sided Wilcoxon tests with Bonferroni multiple test correction. We used box plots to present the population proportions by group, where the band in the box plot indicates the median, the box indicates the first and third quartiles and the whiskers indicate ± 1.5× interquartile range.

Differential expression analysis (Extended)

To perform differential expression analyses, we created pseudobulk expression matrices and used linear models as implemented in packages for bulk RNA-seq. For that, the expression matrices were created separately for each cluster, where the gene expression counts per cell were aggregated by scRNA-seq library (a sample in a specific time-point and infection status) using Seurat AggregateExpression function. Libraries with less than ten cells in the cluster were excluded. Most libraries from the LTBI group did not pass this threshold for the lymphocyte clusters due to their low number of cells, which excluded the use of pseudobulk DE analysis in these cells by cluster. In the myeloid cell subset, we performed the analysis for all the clusters except for the two smallest ones (AM.10 and AM.11). For the DE analysis in each cluster, genes were filtered in two steps: i) we excluded genes that were expressed in less than 10% of the cells from the cluster in both groups, and ii) we excluded genes detected in less than 70% of the libraries. Libraries were normalized, scaled, and log2 transformed using edgeR v3.40.2 and Limma v3.54.2 (voom) (8-10).

First, we performed a differential expression analysis of the myeloid clusters between resisters and LTBI samples in the absence of *Mtb* ("baseline resister vs LTBI" analysis). For this analysis, we used all libraries per subject and removed the effects of infection and time-of-incubation by adding *Mtb*-infection status and hours of incubation as covariates in the model. In addition, we adjusted the analysis on the following variables to reduce confounding effects: length of HIV/ART of the patient, sequencing batch, fraction of dead cells during library preparation, number of cells aggregated for the pseudobulk expression. For quality control, principal component analysis (PCA) was calculated per cluster with prcomp function from stats v4.2.2 R package using the top 500 variable genes. Libraries from one BAL collected from a resister participant appeared as an outlier in the principal component analyses from the myeloid cell subpopulations. These libraries were not outliers in the cell-type detection and cell proportions. Review of the pipeline from sample preparation suggested possible BAL cell contamination. Hence, these libraries were excluded from the downstream analyses to avoid artifacts in the DE tests caused by cross-contamination (Supplemental Table 1). The results of the "baseline" analyses were presented as the log2FC of the gene expression between resister and LTBI cells in the absence of *Mtb*. For multiple test correction, we used the Benjamini-Hochberg false discovery rates (FDR). Genes were considered differentially expressed when presenting absolute log2FC > 0.2 and FDR < 0.1.

For the differential expression analyses for the *ex vivo Mtb* challenge ("*Mtb*-response" analysis) from the two p.i. time-points, 6h and 24h, cells from libraries that fell outside of the 5-7h and 22-25h incubation ranges were excluded (Supplemental Table 1). For this analysis, we blocked by subject in the design model to adjust for the inter-individual variability within groups (11). For each cluster, six contrast tests were performed. In four contrasts, we tested the

differential expression of genes in response to the *Mtb* challenge by group at the two time-points. For that, we compared the expression of the non-infected vs the infected libraries by group and time-point: "LTBI (6h)", "Resister (6h)", "LTBI (24h)" and "Resister (24h)". Hence, the results from these contrasts presented the *Mtb*-response log2FC by group and time p.i. where a positive and negative log2FC indicate up-regulation and down-regulation in response to the *Mtb* challenge, respectively. In addition, we performed two contrasts per cluster where we compared the *Mtb*-responses between resisters and LTBI by time-point (interaction analysis): "Resister (6h) vs LTBI (6h)" and "Resister (24h) vs LTBI (24h)". At each time-point, the log2FC from the interaction analyses refer to the $log2FC_{[Resister]}$ minus the $log2FC_{[LTBI]}$. Therefore, a positive log2FC in the interaction indicates $log2FC_{[Resister]} > log2FC_{[LTBI]}$ (stronger up-regulation or weaker down-regulation in resister compared to LTBI *Mtb*-responses), whereas a negative log2FC in the interaction indicates $log2FC_{[Resister]} < log2FC_{[LTBI]}$ (weaker up-regulation or stronger down-regulation in resister compared to LTBI *Mtb*-responses). For multiple test correction based on the different contrasts, we used the StageR FDR (12). Genes were considered differentially expressed when presenting absolute log2FC > 0.2 and FDR < 0.2.

Due to the low number of lymphocytes in LTBI BAL, for a pseudobulk analysis of the T cells, we combined the T cell clusters as $CD4^+$ T (L.0, L.5, L.6 and L.13 – except $CD4^+$ Treg) and $CD8^+$ T cells (L.2-4, L.7-10, L.12 and L.14). For the baseline and *Mtb*-response analyses we used the same models as in the myeloid cells. Additionally, to investigate gene expression at the level of the 19 lymphocyte clusters, we used Seurat FindMarkers function with default settings. This analysis was performed for a small number of selected genes with established effect on *Mtb* resistance or T cell activity, followed by a manual confirmation that the DEG was detected in most of the samples. For the baseline, we compared the expression in the 6h non-infected cells

between the two groups. For the *Mtb*-response analysis, we performed eight pairwise contrasts per cluster aiming to detect infection-triggered gene expression changes in a group and tested group differences of expression after infection. For that, we compared i) non-infected cells from the resister vs LTBI samples by time-point (two contrasts), ii) *Mtb*-infected cells from the resister vs LTBI cells by time-point (two contrasts), and ii) *Mtb*-infected vs non-infected cells by group by time-point (four contrasts). Clusters with less than 10 cells in a specific group and condition were excluded. The analysis was done only if a gene was expressed in > 25% of the cells in at least one of the contrasted group of cells. Nominal *P*-values were calculated using Wilcoxon test as implemented by FindMarkers, and multiple test correction per contrast was done by Benjamini-Hochberg FDR. Absolute log2FC > 0.2 and FDR < 0.2 were used as thresholds. For visualization, we used Seurat functions VlnPlot and DotPlot.

For the GSEA analysis, as the tested genes were ranked based on the log2FC * -log10(*P*-value), the gene-sets presented positive normalized enrichment score (NES) when enriched among the genes with significant positive log2FC in the specific contrast, while gene-sets presented negative NES when enriched among genes with significant negative log2FC in the contrast

Transcription factor activity score

Transcription factor activities were inferred per cell given the list of DEG per myeloid cluster detected in the pseudobulk DE analyses. TF analysis was done using the non-infected cells with 6h of incubation. For that, a Univariate Linear Model (ULM) was used to test the TF activity per cell using decoupleR v2.8 (13). For each cell, we calculated a TF t-score based on the linear

correlation of gene expression and TF-gene interaction weights. For the TF-gene interaction we used CollecTRI (14), a curated collection of TFs and their corresponding targets, and tested activity for TF that had at least five DEG as their targets. To assess TF-activity per myeloid cluster, we calculated the mean t-score and standard deviation from the cells in the cluster. The mean t-scores were calculated separately in the cells from the resisters and LTBI in the absence of *Mtb*. A t-test was then used to evaluate significant differences in mean TF-activity per cluster between cells from the two groups. A Benjamini-Hochberg correction was applied to calculate the FDR for all tested TF and myeloid cell subpopulations. TF displaying FDR < 0.01 and absolute difference of normalized TF-score > 0.2 between the two groups were considered significant. For visualization, heatmaps were created using ComplexHeatmap v2.14 package.

Antibodies used

For CITE-seq of BAL cells we used the TotalSeq™-B Human TBNK Cocktail for surface antigens CD19 (clone HIB19), CD3 (clone UCHT1), CD16 (clone 3G8) , CD4 (clone RPA-T4), CD11c (S-HCL-3), CD56 (clone 5.1H11), CD14 (clone M5E2), CD8 (clone SK1) and CD45 (clone 2D1) (BioLegend, Cat # 399902, RRID AB_2832784). For flow cytometry of PBMC we used BD CD45 (clone 2D1) PerCP (BD Biosciences, Cat # 345809), BD CD19 (clone SJ25C1) APC (BD Biosciences, Cat # 345791) and BD CD14 (clone M0P9) FITC (BD Biosciences, Cat # 345784) for the B cell/monocyte panel and BD CD8 (clone SK1) FITC (BD Biosciences, Cat # 345772), BD CD4 (clone SK3) PE (BD Biosciences, Cat # 345769), BD CD3 (clone SK7) APC (BD Biosciences, Cat # 345767) and BD CD45 (clone 2D1) PerCP (BD Biosciences, Cat # 345809) for the T cell panel.

Supplemental references

1. Hao Y, Hao S, Andersen-Nissen E, Mauck WM, 3rd, Zheng S, Butler A, et al. Integrated analysis of multimodal single-cell data. *Cell.* 2021;184(13):3573-87 e29.
2. Travaglini KJ, Nabhan AN, Penland L, Sinha R, Gillich A, Sit RV, et al. A molecular cell atlas of the human lung from single-cell RNA sequencing. *Nature.* 2020;587(7835):619-25.
3. Fava VM, Bourgey M, Nawarathna PM, Orlova M, Cassart P, Vinh DC, et al. A systems biology approach identifies candidate drugs to reduce mortality in severely ill patients with COVID-19. *Sci Adv.* 2022;8(22):eabm2510.
4. Yang S, Corbett SE, Koga Y, Wang Z, Johnson WE, Yajima M, et al. Decontamination of ambient RNA in single-cell RNA-seq with DecontX. *Genome Biol.* 2020;21(1):57.
5. Wang Z, Yang S, Koga Y, Corbett SE, Shea CV, Johnson WE, et al. Celda: a Bayesian model to perform co-clustering of genes into modules and cells into subpopulations using single-cell RNA-seq data. *NAR Genom Bioinform.* 2022;4(3):lqac066.
6. McGinnis CS, Murrow LM, and Gartner ZJ. DoubletFinder: Doublet Detection in Single-Cell RNA Sequencing Data Using Artificial Nearest Neighbors. *Cell Syst.* 2019;8(4):329-37 e4.
7. Hafemeister C, and Satija R. Normalization and variance stabilization of single-cell RNA-seq data using regularized negative binomial regression. *Genome Biol.* 2019;20(1):296.

8. Robinson MD, McCarthy DJ, and Smyth GK. edgeR: a Bioconductor package for differential expression analysis of digital gene expression data. *Bioinformatics.* 2010;26(1):139-40.

9. Ritchie ME, Phipson B, Wu D, Hu Y, Law CW, Shi W, et al. limma powers differential expression analyses for RNA-sequencing and microarray studies. *Nucleic Acids Res.* 2015;43(7):e47.

10. Law CW, Chen Y, Shi W, and Smyth GK. voom: Precision weights unlock linear model analysis tools for RNA-seq read counts. *Genome Biol.* 2014;15(2):R29.

11. Correa-Macedo W, Fava VM, Orlova M, Cassart P, Olivenstein R, Sanz J, et al. Alveolar macrophages from persons living with HIV show impaired epigenetic response to Mycobacterium tuberculosis. *J Clin Invest.* 2021;131(22).

12. Van den Berge K, Soneson C, Robinson MD, and Clement L. stageR: a general stage-wise method for controlling the gene-level false discovery rate in differential expression and differential transcript usage. *Genome Biol.* 2017;18(1):151.

13. Badia IMP, Velez Santiago J, Braunger J, Geiss C, Dimitrov D, Muller-Dott S, et al. decoupleR: ensemble of computational methods to infer biological activities from omics data. *Bioinform Adv.* 2022;2(1):vbac016.

14. Muller-Dott S, Tsirvouli E, Vazquez M, Ramirez Flores RO, Badia IMP, Fallegger R, et al. Expanding the coverage of regulons from high-confidence prior knowledge for accurate estimation of transcription factor activities. *Nucleic Acids Res.* 2023.
